# Fuling Granule, a Traditional Chinese Medicine Compound, Suppresses Cell Proliferation and TGFβ-Induced EMT in Ovarian Cancer

**DOI:** 10.1371/journal.pone.0168892

**Published:** 2016-12-30

**Authors:** Fangfang Tao, Shanming Ruan, Wenhong Liu, Libin Wang, Yang Xiong, Minhe Shen

**Affiliations:** 1 Department of Immunology and Microbiology, Basic Medical College, Zhejiang Chinese Medical University, Hangzhou, Zhejiang, China; 2 Department of Medical Oncology, The First Affiliated Hospital of Zhejiang Chinese Medical University, Hangzhou, Zhejiang, China; 3 Institute of Stem Cell Research, General Hospital of Ningxia Medical University, Yinchuan, Ningxia, China; 4 School of Pharmacy, Zhejiang Chinese Medical University, Hangzhou, Zhejiang, China; Duke University School of Medicine, UNITED STATES

## Abstract

The compound fuling granule (CFG) is a traditional Chinese drug which has been used to treat ovarian cancer in China for over twenty years. Nevertheless, the underlying molecular mechanism of its anti-cancer effect remains unclear. In this study, microarray data analysis was performed to search differentially expressed genes in CFG-treated ovarian cancer cells. Several cell cycle and epithelial-mesenchymal transition (EMT) related genes were identified. The microarray analyses also revealed that CFG potentially regulates EMT in ovarian cancer. We also found that, functionally, CFG significantly suppresses ovarian cancer cell proliferation by cell cycle arrest, apoptosis and senescence and the AKT/GSK-3β pathway is possibly involved. Additionally, the invasion and migration ability of ovarian cancer induced by TGFβ is significantly suppressed by CFG. In conclusion, our results demonstrated that CFG suppresses ovarian cancer cell proliferation as well as TGFβ1-induced EMT *in vitro*. Finally, we discovered that CFG suppresses tumor growth and distant metastasis in vivo. Overall, these findings provide helpful clues to design novel clinical treatments against cancer.

## Introduction

Ovarian cancer is a common tumor of the female reproductive system. It is reported that ovarian cancer is the fifth most common lethal gynecological malignancy, and is more common among women in the age range of 40 to 79 [1]. It is usually diagnosed at a distant stage (FIGO stage, III and IV), with a five-year survival rate of about 27% [1]. However, for the fifteen percent of patients who are diagnosed at the localized stage, the five–year survival rate can reach 92% [1]. Thus, it is obvious that chemoresistant metastasis is a major cause of death [2–3]. Although tumor metastasis plays a crucial role in the development of ovarian cancer and the decrease of the survival rate of the patients, the underlying mechanism has not been thoroughly explored yet.

The standard treatment for ovarian cancer is based on radical surgery followed by chemotherapy with paclitaxel and cisplatin. Vaughan et al. suggested that quality of life and symptom improvement should be included in response and survival rates as a primary endpoint in clinical trials [4]. Accordingly, more emphasis should be paid on minimizing the drugs side effects. In recent years we have seen an explosion in the understanding of the tumor heterogeneity, which poses considerable challenges for the design of effective drug combinations [5]. Molecular-targeted drugs are also applied in ovarian cancer treatment, but the overall survival rate is similar despite the higher expense.

Traditional Chinese medicine (TCM) has been clinically used in the treatment of diseases in China and other Asian countries for thousands of years. Guizhi Fuling Capsule (also call Guizhi Fuling Wan) has been used for thousands of years as a traditional Chinese remedy for the treatment of gynecological inflammatory diseases, including uterine fibroids, endometriosis and primary dysmenorrhea [6–9]. As indicated by the theory of TCM and our clinical experience, patients with ovarian cancer usually show symptoms of blood stasis, and Guizhi Fuling Capsule can promote blood circulation [10]. Based on 20 years of clinical practice, Guizhi Fuling Capsule has been developed into Compound Fuling Granule (CFG) for the treatment of ovarian cancer by the Oncology Department of the Zhejiang Provincial Hospital of Traditional Chinese Medicine in Zhejiang, China. The CFG consists of four ingredients, namely Aconitum napellus (monkshood), Wolfiporia extensa (Peck) Ginns (formerly known as Poria cocos F.A. Wolf) or fuling, Patrinia heterophylla DC (diversifolious patrinia) root, and Radix paeoniae Rubra (red peony root). Among these compounds, aconitine and oleanolic acid have been suggested as chemical markers for quality assessment of CFG [11].Clinically, the CFG has been found to increase chemotherapy efficacy, lower toxicity, reduce the incidence of distant metastasis and drug resistance, and improve the quality of life. In this study, we identified various CFG-regulated cell cycle and EMT genes by microarray analyses. Moreover, functionally we found that CFG can suppress ovarian cancer cell proliferation and EMT *in vitro*. Meanwhile, *in* vivo experiments also revealed that oral administration of CFG inhibits tumor growth and metastasis to lung. In addition, we preliminarily studied the mechanisms underlying the effect of CFG in suppressing ovarian cancer cell proliferation as well as TGFβ1-induced EMT in vitro.

## Results

### CFG regulates cell proliferation- and migration-related genes

**Fig 1:**
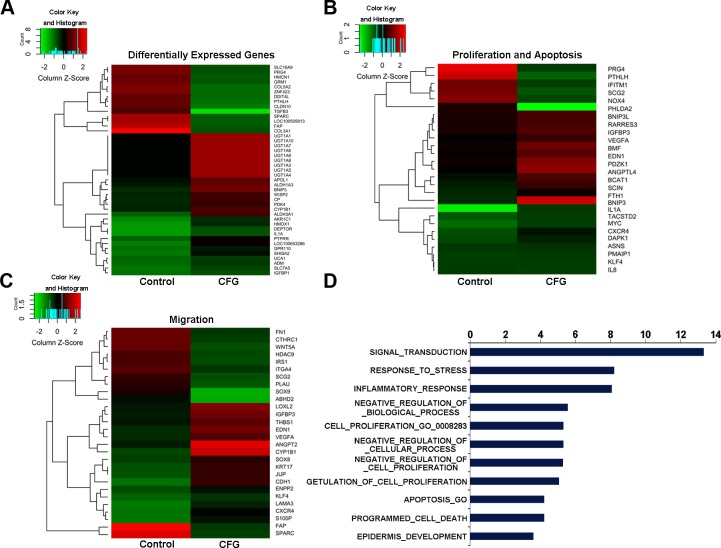
Hierarchical clustering analyses and heatmaps to show differentially regulated gene expression in SKOV3 cells. SKOV3 cells were treated with 10 ng/ml TGFβ1 with or without 3 mg/ml CFG for 24 h. (A) 43 significantly up-regulated and down-regulated genes were identified in the gene expression profiles. 27 cellular proliferation or apoptosis associated genes (B) and 28 EMT-related genes (C) were significantly differentially regulated in the CFG-treated cells. Each gene is presented as the mean ± SD of three independent experiments. (D) Gene Ontology Analysis to indicate the biological process regulated by CFG.

To study the genes differentially regulated by CFG, we performed microarray analysis of CFG-treated SKOV3 cells compared with untreated cells. The number of gene transcripts changed at least 1.5-fold in the CFG-treated cells was 329, of which 216 were from significantly upregulated genes and 113 were from significantly down-regulated genes. Among these we selected 43 key regulated genes which are changed over than 2.5-fold shown in Fig 1A for additional analyses. Further Gene Ontology Analysis (Fig 1D) revealed that lots of genes are involved in the processes of cellular proliferation and apoptosis (Fig 1B) and migration (Fig 1C). Meanwhile, we searched the PubMed database for articles on each of the differentially regulated genes, and reviewed these articles to determine whether the genes were pro-proliferative, anti-proliferative, pro-cell -migration or anti-cell -migration. Explicitly, among these genes, 15 (55.56%) were reported to suppress proliferation, and 12 (44.44%) to promote proliferation (Fig 1B). Additionally, 17 (60.71%) genes were reported to suppress cell migration, and 11 (39.29%) to promote cell migration (Fig 1C). Overall, the microarray data suggest that CFG suppresses cell proliferation and migration *in vitro*.

### CFG inhibits cell proliferation *in vitro*

**Fig 2:**
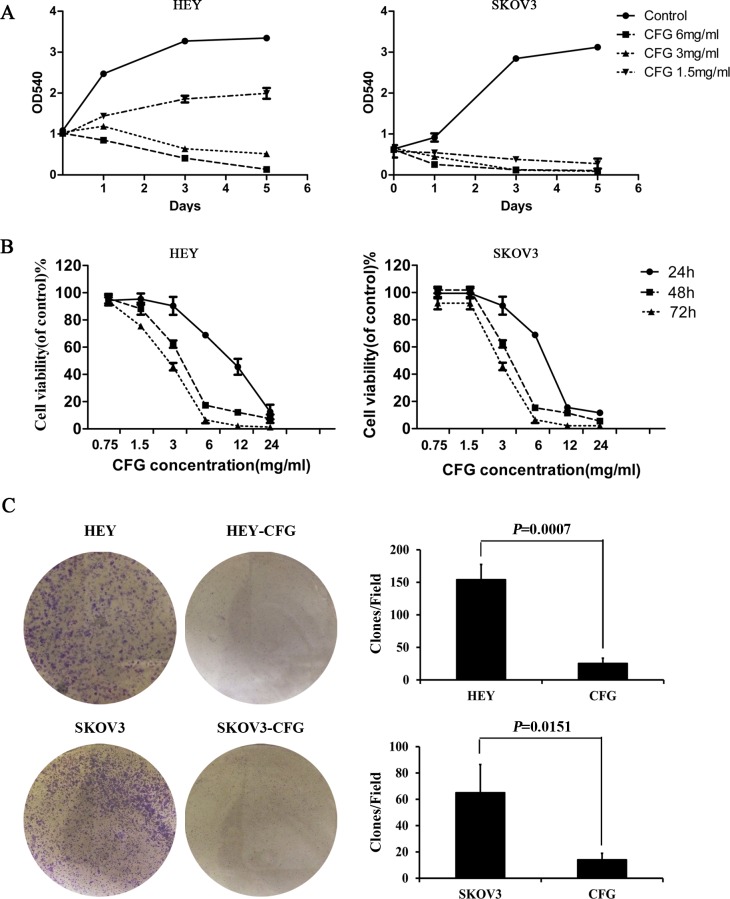
CFG suppresses ovarian cancer cell proliferation *in vitro*. SRB assays (A) and MTT experiments (B) to show the inhibitory effect of CFG on HEY and SKOV3 cell growth after treatment with different concentration of CFG. (C) Crystal violet staining to show the colony formation ability of CFG-treated HEY and SKOV3 cells compared with control. Representative images are presented in the panel. The data represent mean ± SD of three experiments.

To confirm that CFG suppresses cell proliferation in vitro, we used the SRB staining assay to determine the proliferation rates of the n ovarian cancecdr cell lineof doing this and more commonly used is to use the freely available platform from the set up to CFG-treated human ovarian cancer cell lines HEY and SKOV3. We found that, when these cells were treated with different concentrations of CFG (1.5, 3 and 6 mg/ml), their proliferation was significantly suppressed after only one day of treatment. The effect of CFG was more pronounced in SKOV3 cells than in HEY cells, which suggests that SKOV3 cells were more sensitive to CFG than HEY cells (Fig 2A). This phenomenon was also confirmed by the MTT assay (Fig 2B). For HEY cells the IC50 values of CFG were 7.819 mg/ml with 24 h treatment, 3.744 mg/ml for 48 h, and 2.982 mg/ml for 72 h, and for SKOV3 cells, these values were 10.27, 3.709, and 2.684, respectively, suggesting HEY cells were more sensitive to CFG under short-time treatment.

We also examined whether the CFG inhibitory effect on HEY and SKOV3 cells resulted from the killing of the stem-like cancer cells. For this purpose we used the soft agar assay for colony formation to determine the percentages of cancer stem-like cells in the CFG-treated HEY and SKOV3 cells compared with control cells. We found that the colony number was significantly reduced both in HEY and SKOV3 cells after treatment with CFG (Fig 2C).

### CFG affects ovarian cancer cell cycle, apoptosis and senescence in a cell type-dependent manner

**Fig 3:**
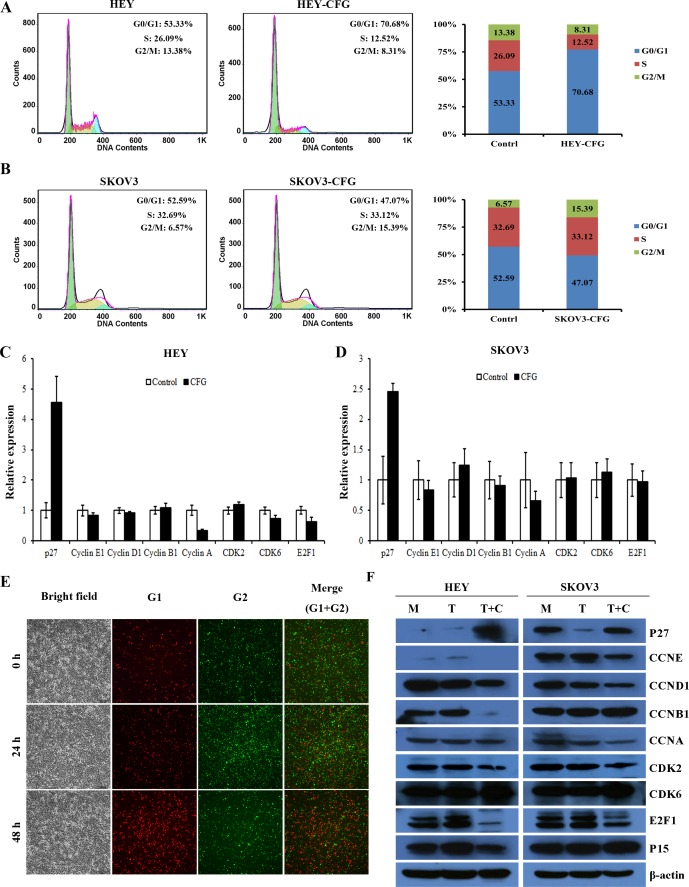
CFG changes cell cycle distribution and cell cycle-related protein expression in ovarian cancer cell lines *in vitro*. HEY (A) and SKOV3 (B) cell lines were treated with CFG 3 mg/ml for 24 h and the cell cycle distribution was analyzed by flow cytometry. (C and D) Quatitative PCR to detect the mRNA expression of cell cycle assoicated protein. HEY cells (C) and SKOV3 cells (D) were treated with 3 mg/ml CFG for 24 h. (E) HEY cells were treated with 3 mg/ml CFG for 24h and 48 h and then stained with immunofluorescen labeled antibodies against Cdt1 (red, G1 phase) and Geminin (green, G2 phase). Representative images are presented. (F) Cell cycle-related proteins were detected by western blot in HEY and SKOV3 cells treated with 3 mg/ml CFG for 24h.

To investigate how the CFG inhibits cell proliferation in vitro, we checked the cell cycle, apoptosis and senescence status of HEY and SKOV3 cells after treatment with CFG. The cell cycle distribution of CFG-treated HEY and SKOV3 cells, determined by flow cytometry analysis, indicated that the percentage of S + G2/M was greatly decreased in HEY cells after CFG treatment, whereas in SKOV3 cells it was actually slightly upregulated (Fig 3A and 3B). Quantitative PCR also showed that the cell cycle associated proteins were regulated by CFG, and this phenomenon was more obvious in HEY than in SKOV3 cells (Fig 3C and 3D). Similarly, immunofluorescence staining of HEY cells with antibodies against Cdt1 (red, G1 phase) and Geminin (green, G2 phase) revealed that proportion of cells in the G2 phase was also reduced after CFG treatment (Fig 3E). Furthermore, the analysis of the cell cycle related proteins by Western blotting showed that the expression of these proteins was indeed significantly changed in CFG treated cells (Fig 3F).

**Fig 4:**
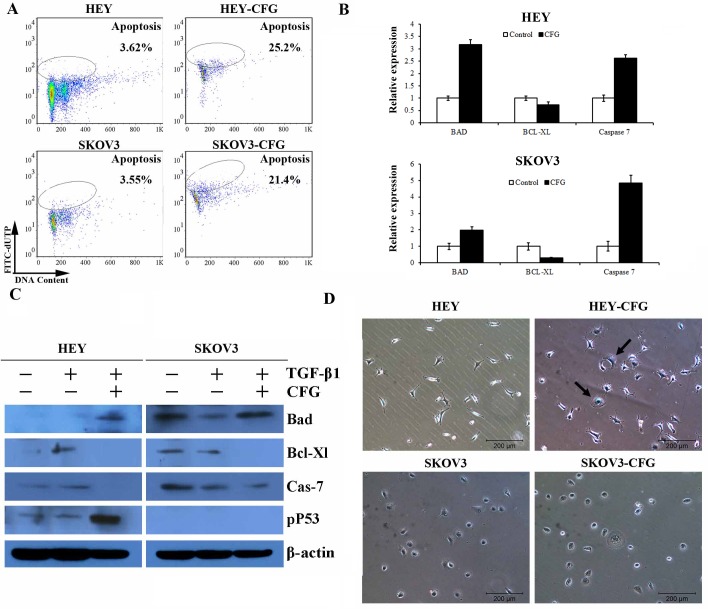
CFG induces apoptosis and senescence in ovarian cancer cells in vitro. (A) HEY and SKOV3 cell lines were treated with 3 mg/ml CFG for 24 h. The percentage of apoptotic cells were measured by flow cytometry using the tunnel apoptosis detection assay. (B and C) Apoptosis-related genes, including Bad, Bcl-Xl, Caspase 7 were detected by quantitative PCR and western blot. (D) β-gal Staining was performed to demonstrate the effect of 3 mg/ml CFG treatment on HEY and SKOV3 cell senescence.

Analysis of apoptosis by cytometry using the tunnel assay indicated that CFG could induce HEY and SKOV3 cell apoptosis (Fig 4A). Moreover, the expression of apoptosis-related proteins, such as p53, Bad, Bcl-XL and Caspase 7 was greatly changed in CFG-treated HEY and SKOV3 cells both in RNA (Fig 4B) and protein levels (Fig 4C). Meanwhile, β-gal staining showed that CFG could induce cellular senescence in HEY cells only (Fig 4D).

### CFG suppresses ovarian cancer cell invasion and migration *in vitro*

**Fig 5:**
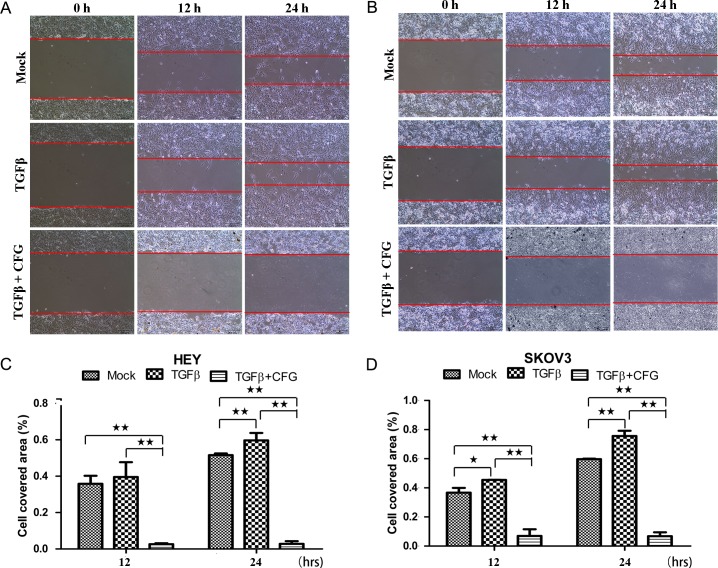
CFG inhibits the mobility of HEY and SKOV3 cells. (A and B) The wound healing assay was used to demonstrate the migration ability of HEY and SKOV3 cells, treated with 3mg/ml CFG only or in combination with 10 ng/ml TGF β1. Cell migration rate was quantitatively evaluated by measuring the cell covered area (C and D). Data are presented as mean ± SD. The experiments were repeated at least three times. *p <0.05 compared with the control. **p <0.01 compared with the control.

**Fig 6:**
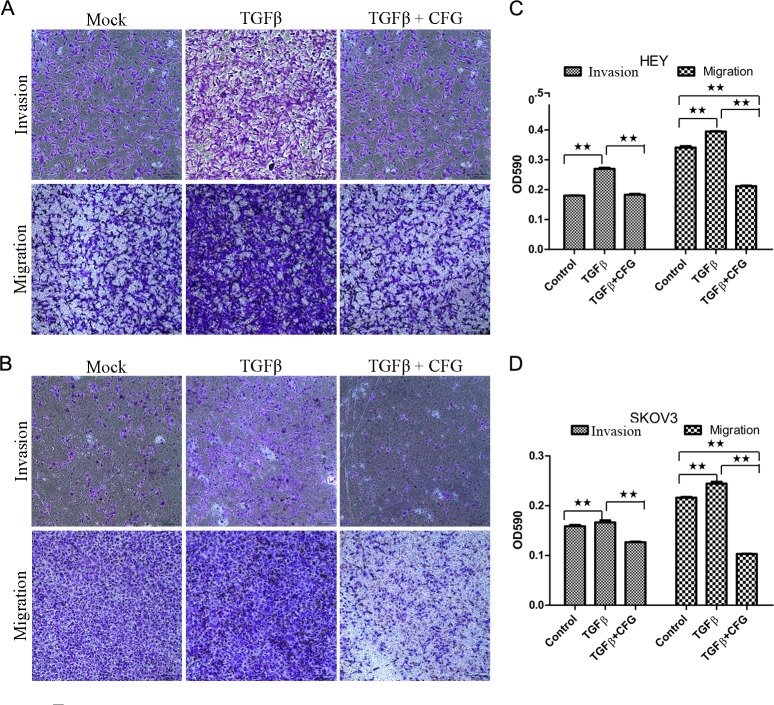
CFG decreases TGF- β 1-induced invasion and migration of HEY and SKOV3 cells in vitro. HEY cells (A) and SKOV3 cells (B) were treated with 3mg/ml CFG only or in combination with 10 ng/ml TGF β1 for 24 h prior to use and the invasion and migration assays were then performed. Crystal violet OD values represent the amounts of invated and migrated HEY cells (C) and SKOV3 cells (D). Data is presented as mean ± SD. The experiments was repeated at least three times. * *p* <0.05 compared with the control. **p <0.01 compared with the control.

In order to determine whether CFG could suppress the invasion and migration ability of the HEY and SKOV3 ovarian cancer cells in vitro, we subjected these cells to the wound healing assay to assess the cellular mobility after treatment with 3 mg/ml of CFG. The results of this test showed that the mobility of both of these ovarian cancer cells was significantly suppressed (Fig 5A, 5B, 5C and 5D). Additionally, we also examined cell invasion and migration by a transwell invasion and migration assay. As anticipated, these results also showed that the invasion and migration ability of ovarian cancer cells was significantly reduced by CFG (Fig 6A, 6B, 6C and 6D).

### CFG down-regulates the expression of EMT markers and the AKT/GSK3β signaling pathway

**Fig 7:**
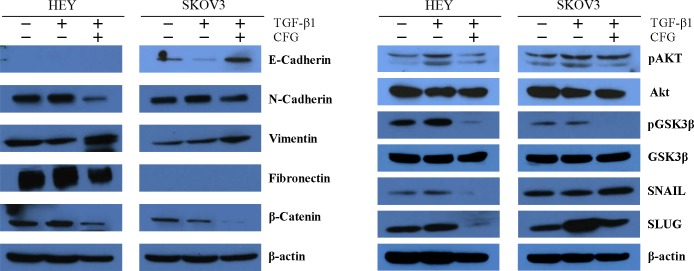
CFG downregultates AKT/GSK3β signal pathway and EMT markers. Cells were treated with 3 mg/ml CFG only or in combination with 10 ng/ml TGF β1 for 24 h. The cellular proteins, including E-cadherin, N-Cadherin, Vimentin, Fibronectin, β-Catenin, pAKT, AKT, pGSK3β, GSK3β, SNAIL, and SLUG, were detected by Western blot.

Since CFG was able to functionally suppress ovarian cancer invasion and migration, we investigated the protein expression of EMT markers, such as E-cadherin, N-Cadherin, Vimentin, and Fibronectin. Consistent with the results of the invasion and migration experiments, CFG reduced the expression of N-Cadherin, Fibronectin and β-Catenin, whereas it increased the expression of E-cadherin. As the EMT is related to the activation of the AKT/GSK3β signaling pathway. We evaluated the potential attenuation of this pathway by CFG treatment by Western blot analysis (Fig 7). The results revealed a specific reduction in the level of pAKT protein in HEY and SKOV3 cells treated with CFG, compared with that of untreated cells, as well as with that of cells singly treated with TGFβ1, as controls. The total AKT level, however, remained unaffected by all treated conditions. The expression levels of AKT downstream substrates Bcl-Xl, BAD, GSK3β, SNAIL, and SLUG were also assessed. CFG treatment also decreased the Bcl-Xl/BAD complex, pGSK3β and SLUG without affecting the nonphosphorylated form of GSK3. Altogether, these data suggested that the regulation of the AKT/GSK3β pathway is associated with the CFG-induced growth inhibition, apoptosis and G2 arrest.

### CFG significantly reduces ovarian tumor formation and suppresses ovarian tumor growth as well as distant metastasis *in vivo*

**Fig 8:**
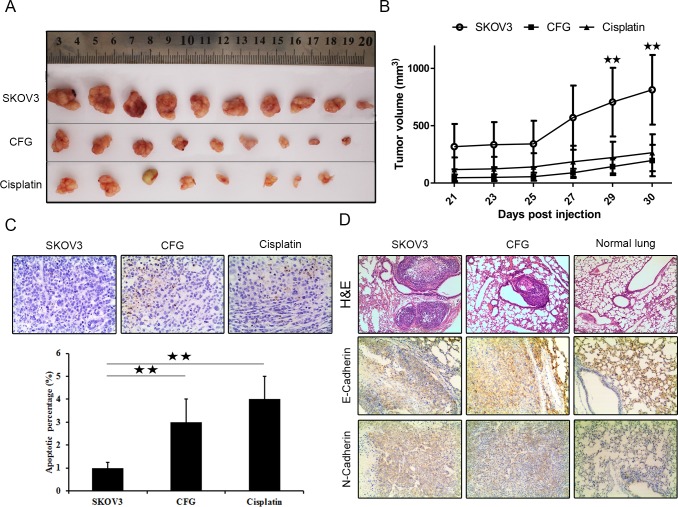
CFG treatment reduced tumor growth and metastasis in vivo xenograft mouse. (A) The photos of the tumor tissues dissected from the mice on day 44 after injection and with aforementioned treatment. (B) The tumor dynamic growth curves of different groups. (C) Tunel assay to show the apoptotic cells in the tumor tissues with indicated treatment at 200× magnification. Lower layer shows the percentage of apoptotic cells in each group. For A, B, and C, SKOV3: SKOV3 control group of subcutaneous injection; CFG: CFG group of subcutaneous injection; Cisplatin: Cisplatin group of subcutaneous injection. (E) H&E staining showed the lung metastases and immunochemistry staining to show the expression of E-Cadherin and N-Cadherin in each group. SKOV3: SKOV3 control group of tail intravenous injection; CFG: CFG group of tail intravenous injection; Normal lung: Normal group for tail intravenous injection. *p < 0.05 and **p < 0.01

Because CFG exhibited significant effects on the proliferation and on the migratory and invasion of ovarian cancer cells *in vitro*, we sought to determine whether CFG could affect the establishment and progression of ovarian cancer *in vivo* nude mouse model. The image of the tumors in Fig 8A were taken from the mice subcutaneously injected with ovarian cancer SKOV3 with aforementioned treatment and the tumor tissues were dissected on day 44. Fig 8B showed the growth curves of the tumors. Both of them revealed that CFG inhibited ovarian tumor growth *in vivo*. Next, we determined the status of apoptosis in these tumors with tunel staining assay and found that CFG and cisplatin obviously promoted tumor cell apoptosis *in vivo* (Fig 8C). We also tail intravenously injected the tumor cells with aforementioned treatment into nude mice and found that less metastases were formed in CFG-treated group (Fig 8D) and immunohistochemistry staining also indicated that CFG treatment led to a marked reduction of N-Cadherin and increase of E-Cadherin expression (Fig 8D).

## Discussion

Ovarian cancer represents the most lethal tumor type among malignancies of the female reproductive systems. The five-year survival rate of distant stage has changed little in the past several decades and most of the patients are diagnosed with a higher stage (III and IV) [1]. These patients have usually been treated by cytoreductive surgery and most of them respond to the first round of chemotherapy. However, some of the patients would undergo a recurrence of chemoresistant tumors in the ovary or other organs. To date, there is no standard treatment for ovarian cancer and the trials of most clinical agents are usually disappointing.

In contrast to Western medicine, TCM does not specially target a single target but to multiple targets involved in complex diseases, including cancer. Recent studies suggest that several of the traditional Chinese medicines and medical therapies are very effective for the treatment or supportive care of tumor patients [12–15]. The management of ovarian cancer and its associated symptoms by TCM approaches has a long history. In recent years, TCM has been used as an adjunct treatment for women with tumor including ovarian cancer as conventional therapies, such as chemotherapy and radiation therapy, may cause severe side effects in the tumor patients.

The available clinical research evidence indicates that CFG is useful to treat ovarian cancer. The aim of this study was to explore the underlying mechanism of the CFG effect on ovarian cancer. Here, we detected alterations in the proliferation, invasion and migration of CFG-treated ovarian cancer cells, which was found to be consistent with the ensuing changes in the expression of various proteins. These results suggest that the CFG could be used on its own to treat ovarian cancer patients In addition, the microarray result and our preliminary experiments, indicated that CFG regulates ovarian cancer cell proliferation and migration via the AKT/GSK3β signaling pathway, which suggests that the CFG may target a molecular component of this pathway. In our *in vivo* tumorigenesis analysis, we found that CFG significantly suppressed tumor formation and tumor burden, which indicated that CFG could work well *in vivo*. In these experiments, we use cisplatin as a positive control. It is interesting that the mice in the cisplatin-treated group obviously lose weight faster than CFG-treated group (data not shown), suggesting that fewer side-effect occurred in the CFG-treated group. Via tail intravenously injection, we also found that less lung metastases formed in CFG-treated group and down-regulation of mesenchymal marker N-cadherin as well as up-regulation of epithelial marker E-Cadherin in CFG-treated group. These results suggest that CFG is functional not only for early-stage ovarian tumors but also for high-grade ovarian tumors.

In order to develop useful therapeutic method for tumor patients, suitable mouse models that mimic the intrinsic biological processes of human cancer tissues are required. During the past several decades, a wide range of ovarian cancer mouse models are available, but the universality of different models is limited for several reasons. Among them, subcutaneous xenograft models are widely used. In this study, both subcutaneous and intravenous injections were used in *in vivo* experiments. However, as a bridge to the clinic, lots of studies [16–19] have shown that implanting human tumor cells orthotopically into the organs of nude mice corresponding to the primary sites in humans results in an increased rate of metastasis in comparison with subcutaneous implantation and orthotopic implantation maybe provide a suitable micro-environment in which ovarian cancer have its intrinsic clinically-relevant properties [16,17,20]. Meanwhile, the orthotopic implantation models have been widely used in several studies on western drugs [21] and traditional Chinese medicines [22–26] as well as bacterial therapies [27–30]. In future studies, we will orthotopically implant human ovarian cancer tissues into nude mice and evaluate the function of CFG in these models.

GSK-3 was reported to be upregulated in various tumor types, including colon, breast, ovarian and pancreatic cancers [31–33], and represents a potential target for therapeutic intervention in cancer [34]. Liu *et al*. found that enhanced AKT activity with subsequent GSK-3β phosphorylation and Snail stabilization, eventually induced epithelial-mesenchymal transition (EMT) and promoted tumor invasion and metastasis [35–36].Our findings are consistent with research indicating its specific role in CFG-induced EMT suppression [37–38]. Indeed, GSK-3β functions in a wide range of tumor cellular processes including cell cycle, proliferation, angiogenesis, invasion and paclitaxel resistant [39–45]. Inhibition of GSK-3 results in cell cycle arrest at the G1 phase and subsequent activation of p27Kip-1 http://www.ncbi.nlm.nih.gov/pmc/articles/PMC4102778/-R64[39]. Increased GSK-3 expression increases the paclitaxel resistance of the ovarian cancer cell line SKOV3 by more than three-fold [44]. Downregulation of Akt/GSK3 and ERK signaling pathways by resveratrol inhibits cell proliferation and the drug resistance of human ovarian cancer cell [45]. These results suggest that the AKT/GSK3 pathway may be a potential molecular mechanism for CFG-suppressed ovarian cancer proliferation.

However, the study on GSK3β remains complex and controversial. GSK-3β also functions as a tumor suppressor, because GSK3β can suppress the Wnt/β-catenin pathway by phosphorylating β-catenin [40]. Moreover, β-catenin transactivation has been associated with cyclin D1 overexpression in breast cancer [46]. In contrast, our study found that CFG both suppress the expression of GSK3β and β-catenin, as well as cyclin D1, which suggests that there may exist more than two monomers in the CFG synergistic targeting of the AKT/GSK3β and Wnt/β-catenin pathways.

In our next study, we plan to extract the monomers of CFG and evaluate their functions in ovarian cancer. At the same time, we also plan to inject this traditional medicine and monomers into mice and study their functions in vivo. Furthermore, we will evaluate the proliferation and metastatic abilities in the PDX models. In human patients, the ovarian cancer usually occurs in different stages. Accordingly, it is very important to evaluate the function in different types and different stages of ovarian cancer. In this study, we found that CFG can suppress the growth of the ovarian cancer cells HEY and SKOV3 in different ways, suggesting that the underlying mechanism is cell type-dependent [47]. As is known, HEY is a serous papillary ovarian carcinoma and SKOV3 is a clear cell carcinoma. Thus, it is important to consider the subtype when treating ovarian cancer. Finally, we also plan to study the function of CFG in other human cancers especially in breast and prostate cancer.

## Materials and Methods

### Cell lines and reagents

Human ovarian cancer cells HEY and SKOV3 were purchased from Cancer Hospital, Chinese Academy of Medical Sciences (Beijing, China). Cells were maintained in DMEM supplemented with 10% fetal bovine serum at 37°C in a humidified 5% CO2 atmosphere. DMEM medium and fetal bovine serum (FBS) were supplied by Gibco-BRL (Rockville, IN, USA). Human TGFβ1 was supplied by PeproTech (NJ, USA). Antibodies against AKT (cat#2920), phospho-AKT [p-AKT (Ser473)] (cat#3787), phospho-GSK3β [p-GSK3β (Ser9)] (cat#9336), GSK3β(cat#9315), phospho-p53 [pP53 (Ser15)] (cat#9284), β-catenin(cat#2849), SLUG(cat#9585), CCNB1(cat#4135), CCNA(cat#4656) were purchased from Cell Signaling Technology (Danvers, MA, USA). Antibodies against CCNE (HE12) (cat#247), CCND1 (HD11) (cat#246), CDK2 (M2) (cat#163), CDK6 (C-21) (cat#177), E2F1 (C-20) (cat#193), P15 (C-20) (cat#612), P27 (F-8) (cat#1641), Geminin (A-3) (cat#74496) and Cdt1(H-300) (cat#28262) were purchased from Santa Cruz Biotechnology. Antibodies against BAD (cat#32445), Bcl-Xl (cat#131870), Caspase-7 (cat#69540), E-cadherin (cat#1416), N-cadherin (cat#12221), Fibronectin (cat#2413), Vimentin (cat#8069), SNAIL (cat#17732) were purchased from Abcam. Antibody against β-actin (cat#A2228) was purchased from Sigma-Aldrich (Saint Louis, MO, USA). In Situ Apoptosis Detection Kit was purchased from Takara (Takara Bio Inc., Shiga, Japan). All other chemicals were purchased from Sigma-Aldrich and Biyuntian Biotech Co., Ltd (Shanghai, China).

### Preparation of CFG ethanol extract

Herbs were supplied by Zhejiang Provincial Hospital of TCM (Zhejiang, China), and identified by Associate Professor Hong Wang, College of Pharmaceutical Sciences, Zhejiang Chinese Medical University. Their Chinese names, English names, Latin names, family, part used, place of origin, voucher number and daily adult doses (g) are presented in Table 1. CFG was prepared as previously described [11]. Briefly, the four constituent herbs were combined in the ratio of 1:1:1:1, and extracted with 75% ethanol (1:10, w/w) twice, 1 h each time, as previously described [11].

**Table 1:** The composition of Compound Fuling Granule (CFG).

Chinese name (Voucher number)	English/Latin name	Family	Part used	Origin
Heifuzi (100111)	Radix aconite Lateralis praeparata/Aconitum carmichaelii Debx.	Ranunculaceae	Processed daughter roots	Sichuan
Mutouhui (100725)	Patrinia heterophylla/Patrinia heterophylla Bge	Valerianaceae	Root	Shanxi
Fuling (110317)	Poria cocos/Poria cocos (Schw.) Wolf	Fungus	Sclerotium	Anhui
Chishao (101218)	Radix paeoniae rubra/Paeonia LactiLora PaLL.	Ranunculaceae	Root	Inner Mongolia

Daily adult dose for each compound: 10 g

### Gene expression profiling and analysis

After serum starvation for 24 h, the ovarian cancer cells were treated with 3 mg/ml CFG only or in combination with 10 ng/ml TGFβ1 for 24 h. Total RNA was extracted from treated and non-treated cell lines using Trizol reagent (Invitrogen, Carlsbad, San Diego, USA) following the manufacturer’s protocol. RNA quality was assessed by the standard of 1.7 < A260/A280 < 2.2 using the Thermo NanoDrop 2000, and also using the standard of RIN > = 7.0 and 28S/18S > 0.7 using the Agilent 2100 Bioanalyzer (Mississauga, Canada). The amplified RNA was prepared with the GeneChip 3’IVT Express Kit (Affymetrix Inc., Santa Clara, CA, USA). After fragmentation, the amplified transcripts were hybridized to an Affymetrix 901838 GeneChip PrimeView Human Gene Expression Array (Affymetrix Inc.) for 16 h in a GeneChip Hybridization Oven 645. Arrays were washed and stained by a GeneChip Fluidics Station 450 and scanned by a GeneChip Scanner 3000. Raw data were imported into GeneSpring GX 11.0.1 and analyzed. Unsupervised hierarchical clustering of genes was applied and heatmaps were constructed by the freely available MeV software: multiExperiments Viewer, which is part of the TM4 Microarray Software Suite (http://www.tm4.org/mev/). The microarray data has been submitted to GEO database and the GEO ID is GSE79454.

### Cell proliferation assay

CFG treated cell proliferation was determined by the sulforhodamine B (SRB) assay. Cells were seeded in 24-well plates and treated with CFG at different concentrations. On the indicated days, cells were fixed with 30% trichloroacetic acid and stained for 30 min at room temperature with 0.4% SRB in 1% acetic acid. The dye was washed off with 1% acetic acid and then dissolved in a 10 mM Tris base solution. The plates were read by a microplate reader (Bio-Rad, Hercules, CA, USA) at 540 nm. Each experiment was conducted in triplicate and repeated at least three times.

In addition, the MTS cytotoxicity assay was also used to monitor cell proliferation. A total of 5×10^3^ cells/well were seeded in 96-well plates in 100 μl culture medium with different concentration of CFG and incubated at 37°C in a humidified 5% CO2 atmosphere for 24 h, 48 h, and 96 h. A 20 μl volume of CellTiter 96 AQueous One Solution Reagent (Promega Corporation, Madison, WI, USA) per well was added and kept incubated as above for 2 h. Then the absorbance was read at 490 nm.

### Cell colony formation

After serum starvation for 24 h, the ovarian cancer cells were treated with 10 ng/ml TGFβ1 only or in combination with 3 mg/ml of CFG for 24 h. Afterwards the treated cells were seeded at approximately 300 cells/well in triplicate into 6-well plates for 2 weeks at 37°C in an atmosphere of 5% CO2. Cells were rinsed twice in PBS and fixed in 4% paraformaldehyde-methanol for 10 min, and stained with 0.1% crystal violet and cell colonies were scored.

### RNA isolation and cDNA analysis by qRT-PCR

qRT-PCR was used to analysis the mRNA expression of P27, CCNE1, CCND1, CCNB1, CCNA, CDK2, CDK6, E2F1, BAD, Bcl-Xl and Cas-7 in tumor cells samples. Total RNA extraction was performed with the RNeasy mini kit (Qiagen) according to the manufacturer’s instructions. Quantitative measurements of different gene levels were obtained using Step One Plus real time PCR (Applied Biosystems) as per the manufacturer’s protocol. The primers were synthesized by HIBIO (Hangzhou, China). The sequences of the primers were as follows: p27: forward primer 5’-TGGAGAAGCACTGCAGAGAC-3’ (20 bp), reverse primer 5’-GCGTGTCCTCAGAGTTAGCC-3’ (20 bp); CCNE1: forward primer 5’-CCACACCTGACAAAGAAGATGATGAC-3’ (26 bp), reverse primer 5’-ACACCTCCATTAACCAATCC-3’ (20 bp); CCND1: forward primer 5’-TATTGCGCTGCTACCGTTGA-3’ (20 bp), reverse primer 5’-CCAATAGCAGCAAACAATGTGAAA-3’ (24 bp); CCNB1: forward primer 5’-TCTGGATAATGGTGAATGGACA-3’ (22 bp), reverse primer 5’-CGATGTGGCATACTTGTTCTTG-3’ (22 bp); CCNA: forward primer 5’-GCACCCTGCTCGTCACTTG-3’ (19 bp), reverse primer 5’-CAGCCCCCAATAAAAGATCCA-3’ (21 bp); CDK2: forward primer 5’-GCTAGCAGACTTTGGACTAGCCAG -3’ (24 bp), reverse primer 5’-AGCTCGGTACCACAGGGTCA-3’ (20 bp); CDK6: forward primer 5’-CTGAATGCTCTTGCTCCTTT-3’ (20 bp), reverse primer 5’-AAAGTTTTGGTGGTCCTTGA-3’ (20 bp); E2F1: forward primer 5’-CACAGATCCCAGCCAGTCTCTA-3’ (22 bp), reverse primer 5’-GAGAAGTCCTCCCGCACATG-3’ (20 bp); BAD: forward primer 5’-CAGGCCTATGCAAAAAGAGGAT-3’ (22bp), reverse primer 5’-CGCACCGGAAGGGAATCT-3’ (18bp); Bcl-Xl: forward primer 5’-CATGGCAGCAGTAAAGCAAG-3’ (20 bp), reverse primer 5’-TAGAGTTCCACAAAAGTATC-3’ (20 bp); Caspase7: forward primer 5’-AGTGACAGGTATGGGCGTTCG-3’ (21 bp), reverse primer 5’-GCATCTATCCCCCCTAAAGTGG-3’ (22 bp); GAPDH: forward primer 5’-GGGACGCTTTCTTTCCTTTC-3’ (20 bp), reverse primer 5’-GCTGCCCATTCATTTCCTT-3’ (20 bp). The experiment was repeated at least once in triplicate.

### Flow cytometric analysis of cell cycle and apoptosis

Cells were collected and fixed with 70% ice-cold ethanol overnight at -20°C. For cell cycle detection, cells were centrifuged, resuspended in a mixture of 50 μg/ml propidium iodide and 10 ng/ml RNase A, and incubated at room temperature for 30 min. Cells were then washed and resuspended in PBS to a final concentration of 1×10^6^/ml, and analyzed with a BD FACS Scalibur (BD Biosciences, San Jose, CA, USA). Apoptosis assay was performed using the APO-direct Apoptosis detection kits (eBiosciences, San Diego, CA, USA), following the standard protocol.

### Senescence-associated-β galactosidase (SA-βgal) assay

Cells were rinsed twice in PBS and fixed at room temperature in 2% formaldehyde/0.2% glutaraldehyde for 15 min. Afterwards cells were washed three times in PBS for 3 min. Cells were then incubated at 37°C overnight (no CO_2_) with fresh SA-β-gal staining solution at pH 6.0: 1 mg of 5-bromo-4-chloro-3-indolyl β-D galactosidase (X-Gal) per ml (stock solution = 20 mg of potassium ferrocyanide/5 mM potassium ferricyanide/150 mM NaCl/2 mM MgCl2) [32]. Cells were washed twice and observed under an ordinary light microscopy. All experiments were performed in triplicate.

### Wound healing assay

Cells were grown to a confluent monolayer in 6-well plates and serum-starved for 24 h prior to use. A wound was inflicted in the cell layer by scratching the plate with a 10 μl sterile pipette tip. Cells were rinsed gently twice with PBS to remove the non-adherent cells before treatment. Cells were then treated with 3 mg/ml CFG only or in combination with 10 ng/ml TGFβ1 and incubated in a reduced-serum medium (1% FBS) for 24 h. Digital images of the wound were acquired at 0 h, 12 h and 24 h. All experiments were performed in triplicate.

### Boyden chamber migration and invasion assays

After treatment with 3 mg/ml CFG only or in combination with 10 ng/ml TGFβ1 and incubation in a reduced serum medium (1% FBS) for 24 h, 2×10^4^ cells were plated into the upper chamber of the transwell membrane (Transwell Permeable Support with a 8.0 μm polycarbonate membrane, 6.5 mm insert, and 24-well plate; Corning Costar, New York, NY, USA) in serum-free medium. Then, medium with 10% FBS was added to the wells. Plates were incubated for 24 h at 37°C in an atmosphere of 5% CO_2_. The bottom cells of the inserts were fixed with 4% paraformaldehyde/cold methanol for 10 min, stained with 0.5% Crystal Violet in 25% methanol for 10 min and rinsed with water to remove excess dye. Membranes were removed from the insert, and the number of cells that migrated through the porous membrane was counted. Invasion assays were performed following the same procedure using the BD BioCoat Matrigel invasion chambers (BD Biosciences). All experiments were performed in triplicate.

### Immunofluorescence

HEY cells were grown on chamber slides and treated with CFG 3 mg/ml for 24 h and 48 h. Cells were fixed with 4% paraformaldehyde, permeabilized with 0.1% Triton X-100, and incubated with the primary antibodies Geminin and Cdt1 separately. Secondary antibodies used were goat anti-mouse IgG-FITC (Santa Cruz Biotechnology, Santa Cruz, CA, USA), and goat anti-rabbit IgG PE (Santa Cruz Biotechnology). Images were acquired using an inverted immunofluorescence microscope (ZEISS Axiovert 200 M, Goettingen, German).

### Western blot analysis

Cells were harvested and boiled in lysis buffer supplemented with complete protease inhibitor cocktail (Roche Inc., Mannheim, Germany) and PMSF. Samples were subjected to SDS-PAGE and transferred to a PVDF membrane. Membranes were incubated with primary antibodies at 4°C overnight, washed with TBST buffer, and incubated again with an appropriate HRP-conjugated secondary antibody at room temperature for 1 h. The membranes were washed and examined by chemiluminescence detection.

### Animals and treatment

All animal experiments were performed with the approval of the Institutional Animal Care and Use Committee of Zhejiang Chinese Medical University and the procedures were in accordance with the guidelines for experimentation with animals (NIH Publication No. 85–23, revised 1996). Animals were maintained in a specific pathogen free laboratory under standard conditions of humility (50 ± 5%), temperature (25 ± 2°C) in a 12 h light/12 h dark cycle and given food and water ad libitum. Female BALB/c (nu/nu) mice (14 ±1 g, 3 weeks old) were purchased from Beijing Vital River Laboratories (Beijing, China) and maintained in our laboratory for one week. Mice were randomly divided into 6 groups, 10 animals for each group. 1) SKOV3 control group of subcutaneous injection. 100 μl PBS was administered by intragatric gavage daily for 44 days. 1×10^7^ SKOV3 cells were inguinal subcutaneous injected once in day 15. 2) CFG group of subcutaneous injection. 30 mg/kg CFG was administered by intragastric gavage daily for 44 days. 1×10^7^ SKOV3 cells were inguinal subcutaneous injected once in day 15. 3) Cisplatin group of subcutaneous injection. 100 μl PBS was administered by intragatric gavage daily for 44 days. 1×10^7^ SKOV3 cells were inguinal subcutaneous injected once in day 15. 3 mg/kg cisplatin was intraperitoneal injected every 3 days starting from 14 days post SKOV3 injection. 4) SKOV3 control group of tail intravenous injection. 100 μl PBS was administered by intragatric gavage daily for 44 days. 1×10^7^ SKOV3 cells were tail intravenous injected in two days from day 15. 5) CFG group of tail intravenous injection. 30 mg/kg CFG was administered by intragastric gavage daily for 44 days. 1×10^7^ SKOV3 cells were tail intravenous injected in two days from day 15. 6) Normal group for tail intravenous injection. 100 μl PBS was administered by intragatric gavage daily for 44 days. To alleviate pain, all mice were anesthetized with isoflurane (5% for induction and 2% for maintenance) before injection with tumor cells. At the euthanasia procedure, the mice were anesthetized by i.p. injection of 100 mg/kg pentobarbital sodium.

### Monitoring of the experimental mice

Mice were inspected every two days and tumor progression was monitored based on overall health and body weight. Size of tumor from subcutaneously injected mice were measured in two dimensions with external calipers every two days from days post SKOV3 injection. Tumor volumes were calculated from calipers measurements with the formula of tumor volume = (W^2^×L) ×1/2 as W is the minor dimension and L is the major dimension of the tumor size. Mice were euthanized and organs (lungs, liver, stomach, kidney, peritoneum, ovaries and spleen) and solid tumors were collected for further examination.

### Histopathological examination and Immunohistochemistry

Tissues were fixed in 4% paraformaldehyde and dehydrated in gradient ethanol. The samples were infiltrated with acetone, absolute xylene and purified paraffin at 65°C for 15 min each, and embedded in paraffin. Sections of 4μm thickness has been taken and stained with hematoxylin and eosin (H&E). For immunohistochemistry staining, 4μm thickness slides were treated with 1mM EDTA buffer (pH = 9.0) in a microwave for 3 min for antigen retrieval. After 3%H_2_O_2_ blocked endogenous peroxidase activity, normal goat serum was used to bind the nonspecific protein. The samples were incubated with primary antibodies. Then slides were incubated with biotin labeled goat-rabbit IgG and horseradish peroxidase-conjugated streptavidin for 1 h, respectively. Immunoreaction was visualized employing diaminobenzidine and counterstained by hematoxylin. Images were taken by an immunofluorescence microscope (ZEISS Axiovert 200 M, Goettingen, German) and representative images were presented.

### Terminal deoxynucleotidyl transferase dUTP nick end labeling (TUNEL)

The TUNEL assay was performed on tumor tissue as a measurement of in situ apoptosis. Previously sectioned and fixed tumor samples were processed using the In Situ Apoptosis Detection Kit (Takara Bio Inc., Shiga, Japan) according to manufacturer’s instructions. Negative controls were also obtained for each sample by omission of incubation with the TUNEL reaction mixture. The image and photographs were obtained under light microscopy.

### Statistical analysis

Data are expressed as mean ± SD. Statistical differences between the groups were evaluated by one-way analysis of variance (ANOVA). The results were analyzed using the SPSS 20.0 software (IBM Corp., Armonk, NY, USA) and P value less than 0.05 was considered statistically significant. Experiments were repeated three times.

## Supporting Information

S1 File 01 GSE79454 and Gene Ontology Analysis
02 in vitro cell data:SRB, MTT, cell clony, cell distribution, wound healing, invasion and migration
03 qPCR data
04 in vivo data
(ZIP)
