# Correction: Fuling Granule, a Traditional Chinese Medicine Compound, Suppresses Cell Proliferation and TGFβ-Induced EMT in Ovarian Cancer

**DOI:** 10.1371/journal.pone.0296410

**Published:** 2023-12-21

**Authors:** Fangfang Tao, Shanming Ruan, Wenhong Liu, Libin Wang, Yang Xiong, Minhe Shen

After publication of this article [1], concerns were raised about Fig 6, specifically:

- In Fig 6A, the “Mock” invasion panel appeared similar to the “TGFβ + CFG” panel.

The authors stated that this was caused by an accidental error during the preparation of the figures and provided underlying data and a replacement panel. The authors also confirmed that the underlying data for all figures in this article were available and provided these to PLOS for review.

With this Correction, the authors provide an updated version of Fig 6 and the original underlying data for this figure in Supporting Information S1 File.

The authors apologize for the error in the published article.

**Fig 6:**
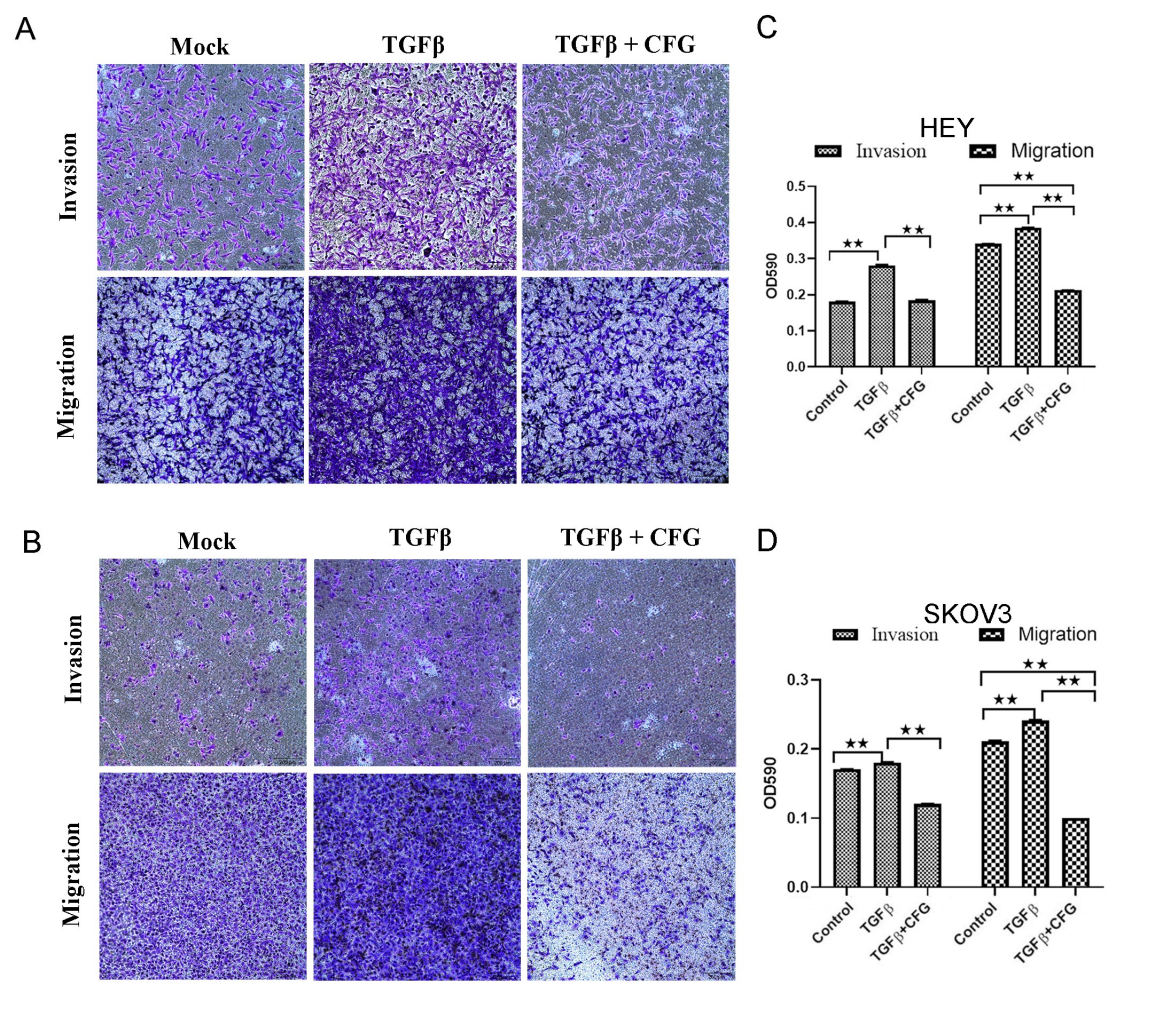
CFG decreases TGF- β 1-induced invasion and migration of HEY and SKOV3 cells in vitro. HEY cells (A) and SKOV3 cells (B) were treated with 3mg/ml CFG only or in combination with 10 ng/ml TGF β1 for 24 h prior to use and the invasion and migration assays were then performed. Crystal violet OD values represent the amounts of invaded and migrated HEY cells (C) and SKOV3 cells (D). Data is presented as mean ± SD. The experiments was repeated at least three times. * *p* <0.05 compared with the control. **p <0.01 compared with the control.

## Supporting information

S1 File Original underlying data for Fig 6. (ZIP)
