# Supplementary material for: Fuling Granule, a Traditional Chinese Medicine Compound, Suppresses Cell Proliferation and TGFβ-Induced EMT in Ovarian Cancer
Source: PLoS One. 2016 Dec 30;11(12):e0168892. doi: 10.1371/journal.pone.0168892 (PMC5201296; doi:10.1371/journal.pone.0168892)
Supplement: S1 File — 01 GSE79454 and Gene Ontology Analysis 02 in vitro cell data:SRB, MTT, cell clony, cell distribution, wound healing, invasion and migration 03 qPCR data 04 in vivo data (ZIP) [file pone.0168892.s001.zip › supporting information/03QPCR data/HEY Cell cycle and Apoptosis QPCR/admin_2016-05-08 17-04-08_CC005309.pdf]

# admin_2016-05-08 17-04-08_CC005309.pcrd

## 5/8/16 07:40 PM

### Report Information

**Experiment Date:** 5/8/16 06:59 PM
**User:** BioRad\admin
**Data File Name:** admin_2016-05-08 17-04-08_CC005309.pcrd
**Data File Path:** F:\ZZQ\20160508
**Selected Well Group:** All Wells

## Experiment Setup

### Run Information

**Run User:** BioRad\admin
**ID:**
**Notes:**
**Sample Volume:** 20
**Temperature Control Mode:** Calculated
**Lid Temperature:** 105
**Lid Force:** AUTO

### Protocol

**1:** 95.0°C for 2:00
**2:** 95.0°C for 0:20
**3:** 59.0°C for 0:25
**4:** 72.0°C for 0:30
Plate Read
**5:** GOTO 2, 35 more times
**6:** Melt Curve 65°C to 95°C : Increment 0.5°C for 0:05
Plate Read

### Plate Display

| | 1 | 2 | 3 | 4 | 5 | 6 | 7 | 8 | 9 | 10 | 11 | 12 |
|---|---|---|---|---|---|---|---|---|---|---|---|---|
| A | Unk<br>p27<br>Control | Unk<br>p27<br>Control | Unk<br>p27<br>Control | Unk<br>p27<br>CFG | Unk<br>p27<br>CFG | Unk<br>p27<br>CFG | | | | | | |
| B | Unk<br>Cyclin E1<br>Control | Unk<br>Cyclin E1<br>Control | Unk<br>Cyclin E1<br>Control | Unk<br>Cyclin E1<br>CFG | Unk<br>Cyclin E1<br>CFG | Unk<br>Cyclin E1<br>CFG | Unk<br>BAD<br>Control | Unk<br>BAD<br>Control | Unk<br>BAD<br>Control | Unk<br>BAD<br>CFG | Unk<br>BAD<br>CFG | Unk<br>BAD<br>CFG |
| C | Unk<br>Cyclin D1<br>Control | Unk<br>Cyclin D1<br>Control | Unk<br>Cyclin D1<br>Control | Unk<br>Cyclin D1<br>CFG | Unk<br>Cyclin D1<br>CFG | Unk<br>Cyclin D1<br>CFG | Unk<br>BCL-XL<br>Control | Unk<br>BCL-XL<br>Control | Unk<br>BCL-XL<br>Control | Unk<br>BCL-XL<br>CFG | Unk<br>BCL-XL<br>CFG | Unk<br>BCL-XL<br>CFG |
| D | Unk<br>Cyclin B1<br>Control | Unk<br>Cyclin B1<br>Control | Unk<br>Cyclin B1<br>Control | Unk<br>Cyclin B1<br>CFG | Unk<br>Cyclin B1<br>CFG | Unk<br>Cyclin B1<br>CFG | Unk<br>Caspase 7<br>Control | Unk<br>Caspase 7<br>Control | Unk<br>Caspase 7<br>Control | Unk<br>Caspase 7<br>CFG | Unk<br>Caspase 7<br>CFG | Unk<br>Caspase 7<br>CFG |
| E | Unk<br>Cyclin A<br>Control | Unk<br>Cyclin A<br>Control | Unk<br>Cyclin A<br>Control | Unk<br>Cyclin A<br>CFG | Unk<br>Cyclin A<br>CFG | Unk<br>Cyclin A<br>CFG | Unk<br>GAPDH<br>Control | Unk<br>GAPDH<br>Control | Unk<br>GAPDH<br>Control | Unk<br>GAPDH<br>CFG | Unk<br>GAPDH<br>CFG | Unk<br>GAPDH<br>CFG |
| F | Unk<br>CDK2<br>Control | Unk<br>CDK2<br>Control | Unk<br>CDK2<br>Control | Unk<br>CDK2<br>CFG | Unk<br>CDK2<br>CFG | Unk<br>CDK2<br>CFG | | | | | | |
| G | Unk<br>CDK6<br>Control | Unk<br>CDK6<br>Control | Unk<br>CDK6<br>Control | Unk<br>CDK6<br>CFG | Unk<br>CDK6<br>CFG | Unk<br>CDK6<br>CFG | | | | | | |
| H | Unk<br>E2F1<br>Control | Unk<br>E2F1<br>Control | Unk<br>E2F1<br>Control | Unk<br>E2F1<br>CFG | Unk<br>E2F1<br>CFG | Unk<br>E2F1<br>CFG | | | | | | |

## Quantitation

**Step #:** 4
**Analysis Mode:** Baseline Subtracted Curve Fit
**Baseline Method per Fluorophore:**
SYBR: Auto Calculated
**Threshold Setting per Fluorophore:**
SYBR: 14.29, Auto Calculated

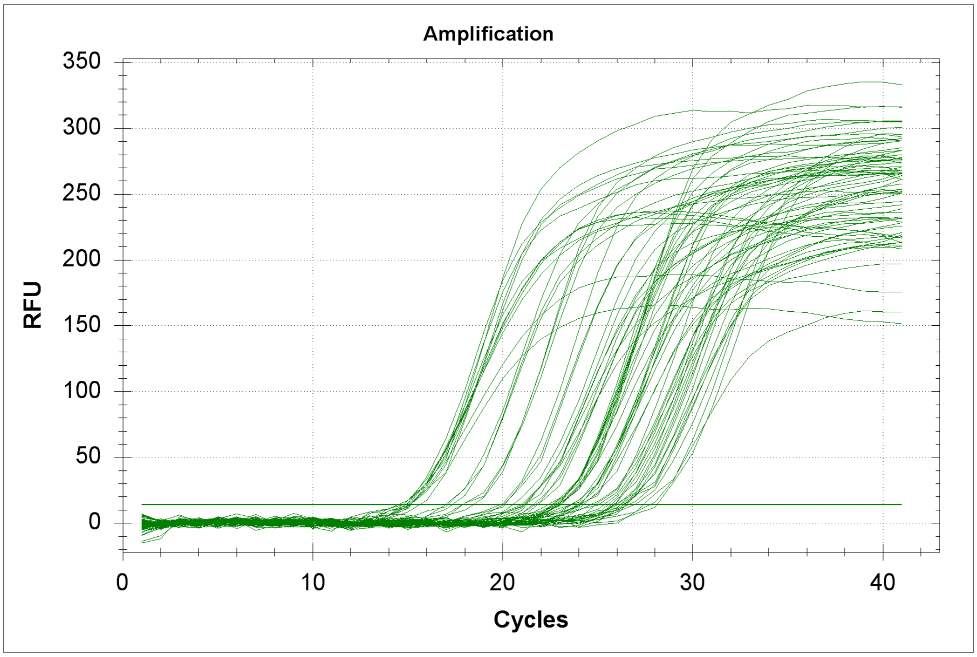


### Quantitation Data

| Well | Fluor | Content | Target | Sample | Threshold Cycle ( C(t) ) | C(t) Mean | C(t) Std. Dev |
|---|---|---|---|---|---|---|---|
| A01 | SYBR | Unkn | p27 | Control | 27.42 | 27.42 | 0.000 |
| A02 | SYBR | Unkn | p27 | Control | 26.34 | 26.34 | 0.000 |
| A03 | SYBR | Unkn | p27 | Control | 26.43 | 26.43 | 0.000 |
| A04 | SYBR | Unkn | p27 | CFG | 25.55 | 25.55 | 0.000 |
| A05 | SYBR | Unkn | p27 | CFG | 24.90 | 24.90 | 0.000 |
| A06 | SYBR | Unkn | p27 | CFG | 24.65 | 24.65 | 0.000 |
| B01 | SYBR | Unkn | Cyclin E1 | Control | 25.33 | 25.33 | 0.000 |
| B02 | SYBR | Unkn | Cyclin E1 | Control | 25.84 | 25.84 | 0.000 |
| B03 | SYBR | Unkn | Cyclin E1 | Control | 25.06 | 25.06 | 0.000 |
| B04 | SYBR | Unkn | Cyclin E1 | CFG | 25.91 | 25.91 | 0.000 |
| B05 | SYBR | Unkn | Cyclin E1 | CFG | 26.22 | 26.22 | 0.000 |
| B06 | SYBR | Unkn | Cyclin E1 | CFG | 26.37 | 26.37 | 0.000 |
| B07 | SYBR | Unkn | BAD | Control | 23.95 | 23.95 | 0.000 |
| B08 | SYBR | Unkn | BAD | Control | 24.10 | 24.10 | 0.000 |
| B09 | SYBR | Unkn | BAD | Control | 24.09 | 24.09 | 0.000 |
| B10 | SYBR | Unkn | BAD | CFG | 22.71 | 22.71 | 0.000 |
| B11 | SYBR | Unkn | BAD | CFG | 22.92 | 22.92 | 0.000 |
| B12 | SYBR | Unkn | BAD | CFG | 23.00 | 23.00 | 0.000 |
| C01 | SYBR | Unkn | Cyclin D1 | Control | 21.14 | 21.14 | 0.000 |
| C02 | SYBR | Unkn | Cyclin D1 | Control | 21.01 | 21.01 | 0.000 |
| C03 | SYBR | Unkn | Cyclin D1 | Control | 21.17 | 21.17 | 0.000 |
| C04 | SYBR | Unkn | Cyclin D1 | CFG | 21.85 | 21.85 | 0.000 |
| C05 | SYBR | Unkn | Cyclin D1 | CFG | 21.58 | 21.58 | 0.000 |
| C06 | SYBR | Unkn | Cyclin D1 | CFG | 21.73 | 21.73 | 0.000 |
| C07 | SYBR | Unkn | BCL-XL | Control | 19.89 | 19.89 | 0.000 |
| C08 | SYBR | Unkn | BCL-XL | Control | 20.04 | 20.04 | 0.000 |
| C09 | SYBR | Unkn | BCL-XL | Control | 20.11 | 20.11 | 0.000 |
| C10 | SYBR | Unkn | BCL-XL | CFG | 20.53 | 20.53 | 0.000 |
| C11 | SYBR | Unkn | BCL-XL | CFG | 21.24 | 21.24 | 0.000 |
| C12 | SYBR | Unkn | BCL-XL | CFG | 21.11 | 21.11 | 0.000 |
| D01 | SYBR | Unkn | Cyclin B1 | Control | 14.83 | 14.83 | 0.000 |
| D02 | SYBR | Unkn | Cyclin B1 | Control | 14.73 | 14.73 | 0.000 |
| D03 | SYBR | Unkn | Cyclin B1 | Control | 15.16 | 15.16 | 0.000 |
| D04 | SYBR | Unkn | Cyclin B1 | CFG | 15.14 | 15.14 | 0.000 |
| D05 | SYBR | Unkn | Cyclin B1 | CFG | 15.64 | 15.64 | 0.000 |
| D06 | SYBR | Unkn | Cyclin B1 | CFG | 15.04 | 15.04 | 0.000 |
| D07 | SYBR | Unkn | Caspase 7 | Control | 24.06 | 24.06 | 0.000 |
| D08 | SYBR | Unkn | Caspase 7 | Control | 23.59 | 23.59 | 0.000 |
| D09 | SYBR | Unkn | Caspase 7 | Control | 23.98 | 23.98 | 0.000 |
| D10 | SYBR | Unkn | Caspase 7 | CFG | 23.02 | 23.02 | 0.000 |
| D11 | SYBR | Unkn | Caspase 7 | CFG | 23.08 | 23.08 | 0.000 |

| | | | | | | | |
|---|---|---|---|---|---|---|---|
| D12 | SYBR | Unkn | Caspase 7 | CFG | 22.85 | 22.85 | 0.000 |
| E01 | SYBR | Unkn | Cyclin A | Control | 24.25 | 24.25 | 0.000 |
| E02 | SYBR | Unkn | Cyclin A | Control | 23.65 | 23.65 | 0.000 |
| E03 | SYBR | Unkn | Cyclin A | Control | 23.64 | 23.64 | 0.000 |
| E04 | SYBR | Unkn | Cyclin A | CFG | 25.71 | 25.71 | 0.000 |
| E05 | SYBR | Unkn | Cyclin A | CFG | 26.05 | 26.05 | 0.000 |
| E06 | SYBR | Unkn | Cyclin A | CFG | 25.84 | 25.84 | 0.000 |
| E07 | SYBR | Unkn | GAPDH | Control | 14.88 | 14.88 | 0.000 |
| E08 | SYBR | Unkn | GAPDH | Control | 14.52 | 14.52 | 0.000 |
| E09 | SYBR | Unkn | GAPDH | Control | 14.53 | 14.53 | 0.000 |
| E10 | SYBR | Unkn | GAPDH | CFG | 15.18 | 15.18 | 0.000 |
| E11 | SYBR | Unkn | GAPDH | CFG | 15.16 | 15.16 | 0.000 |
| E12 | SYBR | Unkn | GAPDH | CFG | 15.07 | 15.07 | 0.000 |
| F01 | SYBR | Unkn | CDK2 | Control | 22.79 | 22.79 | 0.000 |
| F02 | SYBR | Unkn | CDK2 | Control | 22.60 | 22.60 | 0.000 |
| F03 | SYBR | Unkn | CDK2 | Control | 22.40 | 22.40 | 0.000 |
| F04 | SYBR | Unkn | CDK2 | CFG | 23.02 | 23.02 | 0.000 |
| F05 | SYBR | Unkn | CDK2 | CFG | 22.85 | 22.85 | 0.000 |
| F06 | SYBR | Unkn | CDK2 | CFG | 22.65 | 22.65 | 0.000 |
| G01 | SYBR | Unkn | CDK6 | Control | 17.35 | 17.35 | 0.000 |
| G02 | SYBR | Unkn | CDK6 | Control | 16.99 | 16.99 | 0.000 |
| G03 | SYBR | Unkn | CDK6 | Control | 17.13 | 17.13 | 0.000 |
| G04 | SYBR | Unkn | CDK6 | CFG | 18.46 | 18.46 | 0.000 |
| G05 | SYBR | Unkn | CDK6 | CFG | 17.89 | 17.89 | 0.000 |
| G06 | SYBR | Unkn | CDK6 | CFG | 17.91 | 17.91 | 0.000 |
| H01 | SYBR | Unkn | E2F1 | Control | 26.64 | 26.64 | 0.000 |
| H02 | SYBR | Unkn | E2F1 | Control | 26.53 | 26.53 | 0.000 |
| H03 | SYBR | Unkn | E2F1 | Control | 26.20 | 26.20 | 0.000 |
| H04 | SYBR | Unkn | E2F1 | CFG | 28.12 | 28.12 | 0.000 |
| H05 | SYBR | Unkn | E2F1 | CFG | 27.77 | 27.77 | 0.000 |
| H06 | SYBR | Unkn | E2F1 | CFG | 26.95 | 26.95 | 0.000 |

## Melt Curve

**Step #:** 6
**Threshold bar settings:**

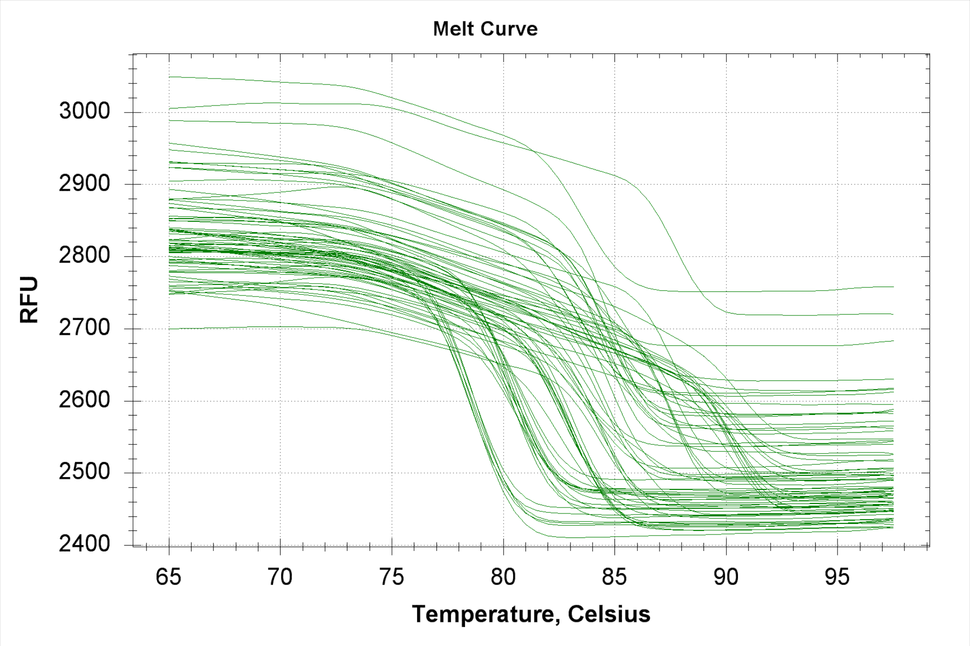

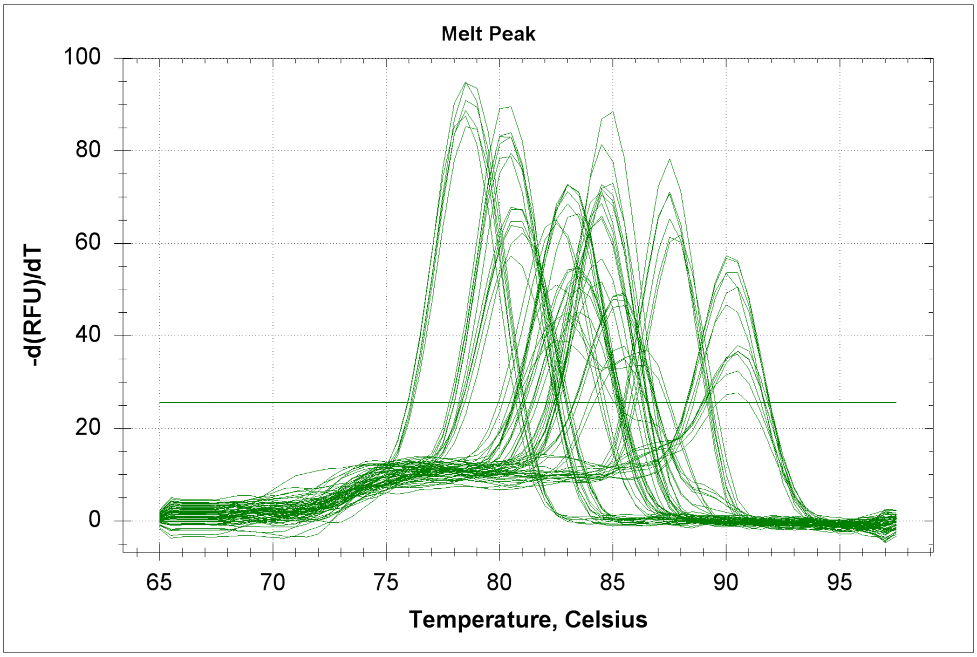


### Melt Curve Data

| Well | Fluor | Content | Sample | Melt Temp |
|---|---|---|---|---|
| A01 | SYBR | Unkn | Control | 90.50 |
| A02 | SYBR | Unkn | Control | 90.50 |
| A03 | SYBR | Unkn | Control | 90.50 |
| A04 | SYBR | Unkn | CFG | 90.50 |
| A05 | SYBR | Unkn | CFG | 90.50 |
| A06 | SYBR | Unkn | CFG | 90.50 |
| B01 | SYBR | Unkn | Control | 84.50 |
| B02 | SYBR | Unkn | Control | 84.50 |
| B03 | SYBR | Unkn | Control | 84.50 |
| B04 | SYBR | Unkn | CFG | 84.50 |
| B05 | SYBR | Unkn | CFG | 84.50 |
| B06 | SYBR | Unkn | CFG | 84.50 |
| B07 | SYBR | Unkn | Control | 83.50 |
| B08 | SYBR | Unkn | Control | 83.50 |
| B09 | SYBR | Unkn | Control | 83.50 |
| B10 | SYBR | Unkn | CFG | 83.50 |
| B11 | SYBR | Unkn | CFG | 83.50 |
| B12 | SYBR | Unkn | CFG | 83.50 |
| C01 | SYBR | Unkn | Control | 81.00 |
| C02 | SYBR | Unkn | Control | 80.50 |
| C03 | SYBR | Unkn | Control | 80.50 |
| C04 | SYBR | Unkn | CFG | 80.50 |
| C05 | SYBR | Unkn | CFG | 80.50 |
| C06 | SYBR | Unkn | CFG | 81.00 |
| C07 | SYBR | Unkn | Control | 87.50 |
| C08 | SYBR | Unkn | Control | 87.50 |
| C09 | SYBR | Unkn | Control | 87.50 |
| C10 | SYBR | Unkn | CFG | 87.50 |
| C11 | SYBR | Unkn | CFG | 87.50 |
| C12 | SYBR | Unkn | CFG | 88.00 |
| D01 | SYBR | Unkn | Control | 80.50 |
| D02 | SYBR | Unkn | Control | 80.50 |
| D03 | SYBR | Unkn | Control | 80.00 |
| D04 | SYBR | Unkn | CFG | 80.50 |
| D05 | SYBR | Unkn | CFG | 80.50 |
| D06 | SYBR | Unkn | CFG | 80.50 |
| D07 | SYBR | Unkn | Control | 84.50 |
| D08 | SYBR | Unkn | Control | 84.50 |
| D09 | SYBR | Unkn | Control | 84.50 |
| D10 | SYBR | Unkn | CFG | 84.50 |
| D11 | SYBR | Unkn | CFG | 85.00 |
| D12 | SYBR | Unkn | CFG | 85.00 |

| | | | | |
|---|---|---|---|---|
| E01 | SYBR | Unkn | Control | 82.50 |
| E02 | SYBR | Unkn | Control | 82.50 |
| E03 | SYBR | Unkn | Control | 82.50 |
| E04 | SYBR | Unkn | CFG | 83.00 |
| E05 | SYBR | Unkn | CFG | 86.50 |
| E05 | SYBR | Unkn | CFG | 82.50 |
| E06 | SYBR | Unkn | CFG | 86.50 |
| E06 | SYBR | Unkn | CFG | 83.00 |
| E07 | SYBR | Unkn | Control | 85.50 |
| E08 | SYBR | Unkn | Control | 85.00 |
| E09 | SYBR | Unkn | Control | 85.50 |
| E10 | SYBR | Unkn | CFG | 85.50 |
| E11 | SYBR | Unkn | CFG | 85.50 |
| E12 | SYBR | Unkn | CFG | 85.50 |
| F01 | SYBR | Unkn | Control | 83.50 |
| F02 | SYBR | Unkn | Control | 83.00 |
| F03 | SYBR | Unkn | Control | 83.00 |
| F04 | SYBR | Unkn | CFG | 83.00 |
| F05 | SYBR | Unkn | CFG | 83.00 |
| F06 | SYBR | Unkn | CFG | 83.00 |
| G01 | SYBR | Unkn | Control | 78.50 |
| G02 | SYBR | Unkn | Control | 78.50 |
| G03 | SYBR | Unkn | Control | 78.50 |
| G04 | SYBR | Unkn | CFG | 78.50 |
| G05 | SYBR | Unkn | CFG | 78.50 |
| G06 | SYBR | Unkn | CFG | 78.50 |
| H01 | SYBR | Unkn | Control | 90.50 |
| H02 | SYBR | Unkn | Control | 90.00 |
| H03 | SYBR | Unkn | Control | 90.00 |
| H04 | SYBR | Unkn | CFG | 90.00 |
| H05 | SYBR | Unkn | CFG | 90.00 |
| H06 | SYBR | Unkn | CFG | 90.00 |

## Gene Expression

**Analysis Mode:** Normalized expression ( $\Delta\Delta C(t)$ )
**Chart Data:** Relative to zero
**Scaling options:**
**Chart Error:** ±1.0

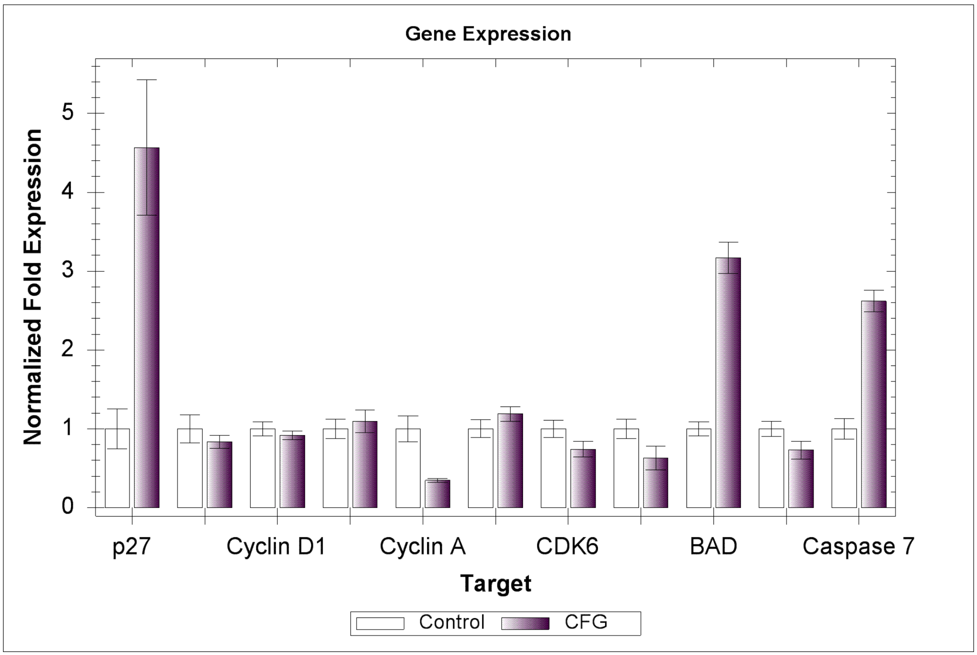


## Target Names

| Name | FullName | Reference | Auto Efficiency | Efficiency |
|---|---|---|---|---|
| p27 | p27 | No | Yes | 100.0% |
| Cyclin E1 | Cyclin E1 | No | Yes | 100.0% |
| Cyclin D1 | Cyclin D1 | No | Yes | 100.0% |

| | | | | |
|---|---|---|---|---|
| Cyclin B1 | Cyclin B1 | No | Yes | 100.0% |
| Cyclin A | Cyclin A | No | Yes | 100.0% |
| CDK2 | CDK2 | No | Yes | 100.0% |
| CDK6 | CDK6 | No | Yes | 100.0% |
| E2F1 | E2F1 | No | Yes | 100.0% |
| BAD | BAD | No | Yes | 100.0% |
| BCL-XL | BCL-XL | No | Yes | 100.0% |
| Caspase 7 | Caspase 7 | No | Yes | 100.0% |
| GAPDH | GAPDH | Yes | Yes | 100.0% |

### Sample Names

| Name | FullName | Control |
|---|---|---|
| Control | Control | Yes |
| CFG | CFG | No |

### Gene Expression Data

| Target | Sample | Ctrl | Expression | Expression SEM | Corrected Expression SEM | Mean C(t) | C(t) SEM |
|---|---|---|---|---|---|---|---|
| BAD | CFG | | 3.17175 | 0.19897 | 0.19897 | 22.88 | 0.08416 |
| BAD | Control | * | 1.00000 | 0.08937 | 0.08937 | 24.05 | 0.05099 |
| BCL-XL | CFG | | 0.73085 | 0.11202 | 0.11202 | 20.96 | 0.21861 |
| BCL-XL | Control | * | 1.00000 | 0.09335 | 0.09335 | 20.01 | 0.06414 |
| Caspase 7 | CFG | | 2.62047 | 0.13743 | 0.13743 | 22.98 | 0.06794 |
| Caspase 7 | Control | * | 1.00000 | 0.13079 | 0.13079 | 23.88 | 0.14690 |
| CDK2 | CFG | | 1.19039 | 0.09203 | 0.09203 | 22.84 | 0.10645 |
| CDK2 | Control | * | 1.00000 | 0.11249 | 0.11249 | 22.60 | 0.11097 |
| CDK6 | CFG | | 0.74127 | 0.09730 | 0.09730 | 18.08 | 0.18643 |
| CDK6 | Control | * | 1.00000 | 0.10963 | 0.10963 | 17.16 | 0.10483 |
| Cyclin A | CFG | | 0.34712 | 0.02525 | 0.02525 | 25.86 | 0.09954 |
| Cyclin A | Control | * | 1.00000 | 0.16246 | 0.16246 | 23.84 | 0.20226 |
| Cyclin B1 | CFG | | 1.09303 | 0.14354 | 0.14354 | 15.27 | 0.18652 |
| Cyclin B1 | Control | * | 1.00000 | 0.12129 | 0.12129 | 14.91 | 0.12881 |
| Cyclin D1 | CFG | | 0.91894 | 0.05350 | 0.05350 | 21.72 | 0.07711 |
| Cyclin D1 | Control | * | 1.00000 | 0.08887 | 0.08887 | 21.11 | 0.04914 |
| Cyclin E1 | CFG | | 0.83490 | 0.08035 | 0.08035 | 26.16 | 0.13480 |
| Cyclin E1 | Control | * | 1.00000 | 0.17755 | 0.17755 | 25.41 | 0.22714 |
| E2F1 | CFG | | 0.63092 | 0.15167 | 0.15167 | 27.62 | 0.34521 |
| E2F1 | Control | * | 1.00000 | 0.12314 | 0.12314 | 26.46 | 0.13243 |
| GAPDH | CFG | | N/A | N/A | N/A | 15.14 | 0.03329 |
| GAPDH | Control | * | N/A | N/A | N/A | 14.64 | 0.11842 |
| p27 | CFG | | 4.56626 | 0.85836 | 0.85836 | 25.03 | 0.26914 |
| p27 | Control | * | 1.00000 | 0.25196 | 0.25196 | 26.73 | 0.34366 |

## End Point

**Fluorophore:** SYBR
**End Cycles to Average:** 5
**Mode:** Percentage of Range - 10
**Lowest RFU Value:** 154
**Highest RFU Value:** 334
**Cut Off Value:**

### End Point Data

| Well | Fluor | Content | Sample | End RFU | Call |
|---|---|---|---|---|---|
| A01 | SYBR | Unkn | Control | 160 | |
| A02 | SYBR | Unkn | Control | 212 | |
| A03 | SYBR | Unkn | Control | 222 | |
| A04 | SYBR | Unkn | CFG | 208 | |
| A05 | SYBR | Unkn | CFG | 210 | |
| A06 | SYBR | Unkn | CFG | 222 | |
| B01 | SYBR | Unkn | Control | 228 | |
| B02 | SYBR | Unkn | Control | 235 | |
| B03 | SYBR | Unkn | Control | 281 | |
| B04 | SYBR | Unkn | CFG | 259 | |
| B05 | SYBR | Unkn | CFG | 195 | |
| B06 | SYBR | Unkn | CFG | 269 | |
| B07 | SYBR | Unkn | Control | 228 | |
| B08 | SYBR | Unkn | Control | 208 | |

| | | | | | |
|---|---|---|---|---|---|
| B09 | SYBR | Unkn | Control | 235 | |
| B10 | SYBR | Unkn | CFG | 239 | |
| B11 | SYBR | Unkn | CFG | 230 | |
| B12 | SYBR | Unkn | CFG | 217 | |
| C01 | SYBR | Unkn | Control | 230 | |
| C02 | SYBR | Unkn | Control | 248 | |
| C03 | SYBR | Unkn | Control | 209 | |
| C04 | SYBR | Unkn | CFG | 242 | |
| C05 | SYBR | Unkn | CFG | 245 | |
| C06 | SYBR | Unkn | CFG | 262 | |
| C07 | SYBR | Unkn | Control | 305 | |
| C08 | SYBR | Unkn | Control | 268 | |
| C09 | SYBR | Unkn | Control | 265 | |
| C10 | SYBR | Unkn | CFG | 265 | |
| C11 | SYBR | Unkn | CFG | 232 | |
| C12 | SYBR | Unkn | CFG | 251 | |
| D01 | SYBR | Unkn | Control | 278 | |
| D02 | SYBR | Unkn | Control | 251 | |
| D03 | SYBR | Unkn | Control | 267 | |
| D04 | SYBR | Unkn | CFG | 288 | |
| D05 | SYBR | Unkn | CFG | 276 | |
| D06 | SYBR | Unkn | CFG | 317 | |
| D07 | SYBR | Unkn | Control | 281 | |
| D08 | SYBR | Unkn | Control | 277 | |
| D09 | SYBR | Unkn | Control | 275 | |
| D10 | SYBR | Unkn | CFG | 315 | |
| D11 | SYBR | Unkn | CFG | 334 | |
| D12 | SYBR | Unkn | CFG | 275 | |
| E01 | SYBR | Unkn | Control | 214 | |
| E02 | SYBR | Unkn | Control | 257 | |
| E03 | SYBR | Unkn | Control | 264 | |
| E04 | SYBR | Unkn | CFG | 247 | |
| E05 | SYBR | Unkn | CFG | 273 | |
| E06 | SYBR | Unkn | CFG | 276 | |
| E07 | SYBR | Unkn | Control | 221 | |
| E08 | SYBR | Unkn | Control | 221 | |
| E09 | SYBR | Unkn | Control | 218 | |
| E10 | SYBR | Unkn | CFG | 213 | |
| E11 | SYBR | Unkn | CFG | 178 | |
| E12 | SYBR | Unkn | CFG | 154 | |
| F01 | SYBR | Unkn | Control | 266 | |
| F02 | SYBR | Unkn | Control | 289 | |
| F03 | SYBR | Unkn | Control | 298 | |
| F04 | SYBR | Unkn | CFG | 292 | |
| F05 | SYBR | Unkn | CFG | 274 | |
| F06 | SYBR | Unkn | CFG | 269 | |
| G01 | SYBR | Unkn | Control | 265 | |
| G02 | SYBR | Unkn | Control | 304 | |
| G03 | SYBR | Unkn | Control | 272 | |
| G04 | SYBR | Unkn | CFG | 295 | |
| G05 | SYBR | Unkn | CFG | 306 | |
| G06 | SYBR | Unkn | CFG | 290 | |
| H01 | SYBR | Unkn | Control | 252 | |
| H02 | SYBR | Unkn | Control | 275 | |
| H03 | SYBR | Unkn | Control | 251 | |
| H04 | SYBR | Unkn | CFG | 282 | |
| H05 | SYBR | Unkn | CFG | 290 | |
| H06 | SYBR | Unkn | CFG | 249 | |
