# Supplementary material for: Fuling Granule, a Traditional Chinese Medicine Compound, Suppresses Cell Proliferation and TGFβ-Induced EMT in Ovarian Cancer
Source: PLoS One. 2016 Dec 30;11(12):e0168892. doi: 10.1371/journal.pone.0168892 (PMC5201296; doi:10.1371/journal.pone.0168892)
Supplement: S1 File — 01 GSE79454 and Gene Ontology Analysis 02 in vitro cell data:SRB, MTT, cell clony, cell distribution, wound healing, invasion and migration 03 qPCR data 04 in vivo data (ZIP) [file pone.0168892.s001.zip › supporting information/03QPCR data/SKOV3 Apoptosis QPCR/admin_2016-05-12 22-05-17_CC005309-2.pdf]

BIO-RAD

admin_2016-05-12 22-05-17_CC005309.pcrd

5/14/16 04:37 PM

Report Information

Experiment Date: 5/12/16 11:43 PM
User: BioRad\admin
Data File Name: admin_2016-05-12 22-05-17_CC005309.pcrd
Data File Path: G:\20160512
Selected Well Group: All Wells

Experiment Setup

Run Information

Run User: BioRad\admin
ID:
Notes:
Sample Volume: 25
Temperature Control Mode: Calculated
Lid Temperature: 105
Lid Force: AUTO

Protocol

1: 95.0°C for 1:00
2: 95.0°C for 0:20
3: 58.0°C for 0:20
4: 72.0°C for 0:20
Plate Read
5: GOTO 2, 39 more times
6: Melt Curve 65°C to 95°C : Increment 0.5°C for 0:05
Plate Read

Plate Display

	1	2	3	4	5	6	7	8	9	10	11	12
A												
B							Unk<br>BAD<br>Control	Unk<br>BAD<br>Control	Unk<br>BAD<br>Control	Unk<br>BAD<br>CFG	Unk<br>BAD<br>CFG	Unk<br>BAD<br>CFG
C							Unk<br>BCL-XL<br>Control	Unk<br>BCL-XL<br>Control	Unk<br>BCL-XL<br>Control	Unk<br>BCL-XL<br>CFG	Unk<br>BCL-XL<br>CFG	Unk<br>BCL-XL<br>CFG
D							Unk<br>Caspase 7<br>Control	Unk<br>Caspase 7<br>Control	Unk<br>Caspase 7<br>Control	Unk<br>Caspase 7<br>CFG	Unk<br>Caspase 7<br>CFG	Unk<br>Caspase 7<br>CFG
E							Unk<br>GAPDH<br>Control	Unk<br>GAPDH<br>Control	Unk<br>GAPDH<br>Control	Unk<br>GAPDH<br>CFG	Unk<br>GAPDH<br>CFG	Unk<br>GAPDH<br>CFG
F												
G												
H												

Quantitation

Step #: 4
Analysis Mode: Baseline Subtracted Curve Fit
Baseline Method per Fluorophore:
SYBR: Auto Calculated
Threshold Setting per Fluorophore:
SYBR: 13.05, Auto Calculated

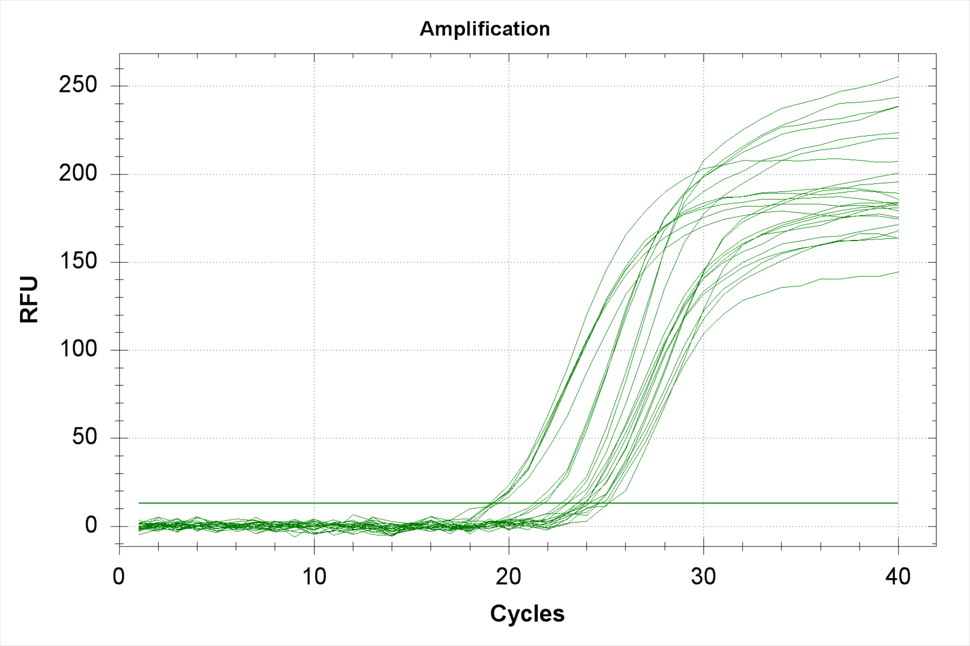

Quantitation Data

Well	Fluor	Content	Target	Sample	Threshold Cycle (C(t))	C(t) Mean	C(t) Std. Dev
B07	SYBR	Unkn	BAD	Control	24.68	24.68	0.000
B08	SYBR	Unkn	BAD	Control	25.09	25.09	0.000
B09	SYBR	Unkn	BAD	Control	24.49	24.49	0.000
B10	SYBR	Unkn	BAD	CFG	23.92	23.92	0.000
B11	SYBR	Unkn	BAD	CFG	23.93	23.93	0.000
B12	SYBR	Unkn	BAD	CFG	23.50	23.50	0.000
C07	SYBR	Unkn	BCL-XL	Control	21.28	21.28	0.000
C08	SYBR	Unkn	BCL-XL	Control	21.77	21.77	0.000
C09	SYBR	Unkn	BCL-XL	Control	21.53	21.53	0.000
C10	SYBR	Unkn	BCL-XL	CFG	23.53	23.53	0.000
C11	SYBR	Unkn	BCL-XL	CFG	24.11	24.11	0.000
C12	SYBR	Unkn	BCL-XL	CFG	23.77	23.77	0.000
D07	SYBR	Unkn	Caspase 7	Control	24.09	24.09	0.000
D08	SYBR	Unkn	Caspase 7	Control	24.59	24.59	0.000
D09	SYBR	Unkn	Caspase 7	Control	25.11	25.11	0.000
D10	SYBR	Unkn	Caspase 7	CFG	22.68	22.68	0.000
D11	SYBR	Unkn	Caspase 7	CFG	23.02	23.02	0.000
D12	SYBR	Unkn	Caspase 7	CFG	22.99	22.99	0.000
E07	SYBR	Unkn	GAPDH	Control	19.12	19.12	0.000
E08	SYBR	Unkn	GAPDH	Control	19.20	19.20	0.000
E09	SYBR	Unkn	GAPDH	Control	19.03	19.03	0.000
E10	SYBR	Unkn	GAPDH	CFG	19.15	19.15	0.000
E11	SYBR	Unkn	GAPDH	CFG	19.32	19.32	0.000
E12	SYBR	Unkn	GAPDH	CFG	19.45	19.45	0.000

Melt Curve

Step #: 6
Threshold bar settings:

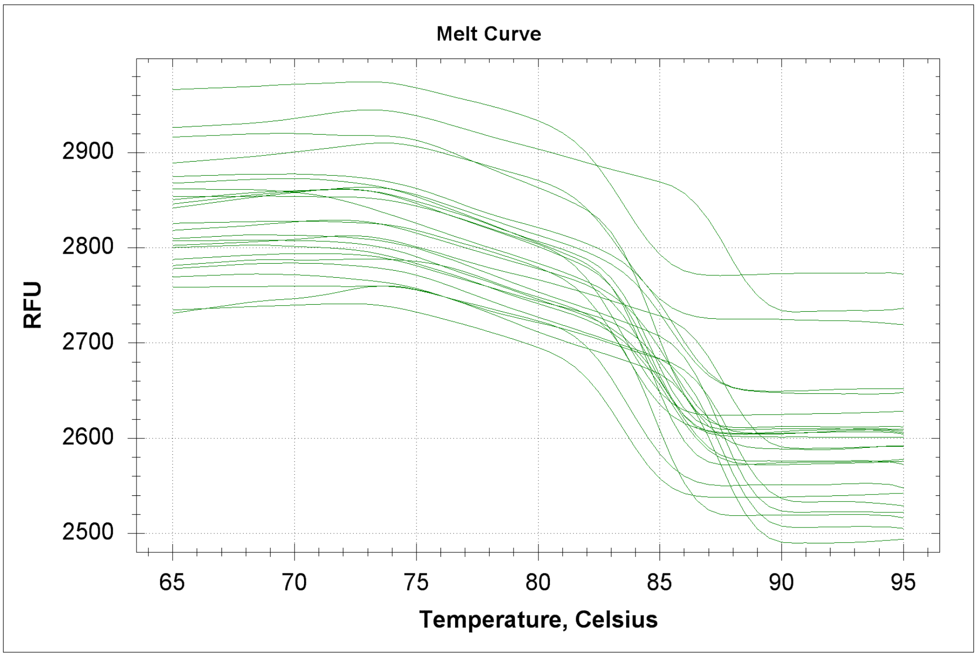

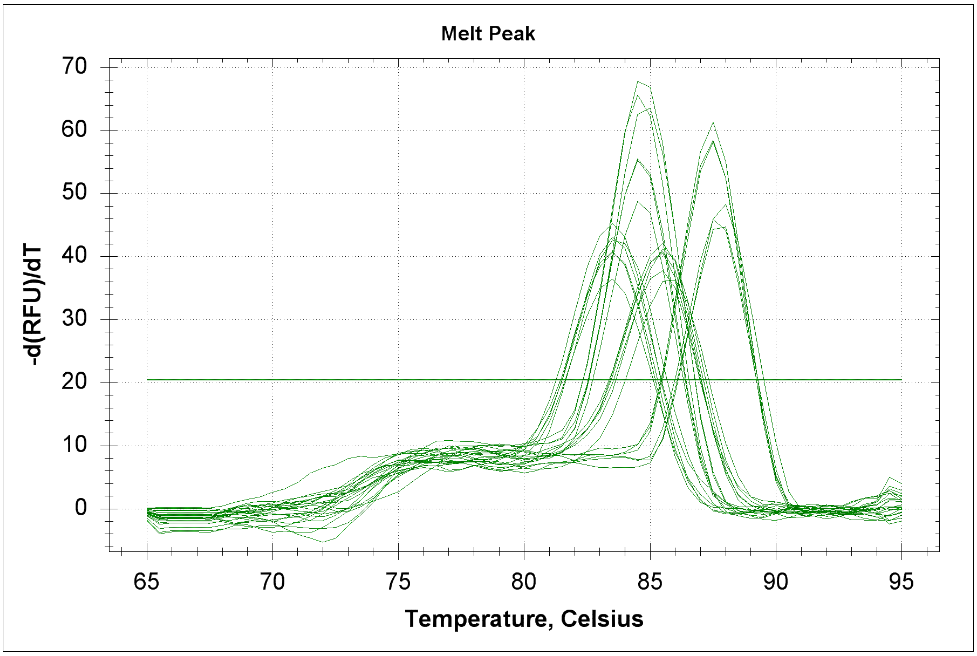

Melt Curve Data

Well	Fluor	Content	Sample	Melt Temp
B07	SYBR	Unkn	Control	83.50
B08	SYBR	Unkn	Control	83.50
B09	SYBR	Unkn	Control	83.50
B10	SYBR	Unkn	CFG	83.50
B11	SYBR	Unkn	CFG	83.50
B12	SYBR	Unkn	CFG	83.50
C07	SYBR	Unkn	Control	87.50
C08	SYBR	Unkn	Control	87.50
C09	SYBR	Unkn	Control	87.50
C10	SYBR	Unkn	CFG	87.50
C11	SYBR	Unkn	CFG	88.00
C12	SYBR	Unkn	CFG	88.00
D07	SYBR	Unkn	Control	84.50
D08	SYBR	Unkn	Control	84.50
D09	SYBR	Unkn	Control	84.50
D10	SYBR	Unkn	CFG	84.50
D11	SYBR	Unkn	CFG	84.50
D12	SYBR	Unkn	CFG	85.00

E07	SYBR	Unkn	Control	85.50
E08	SYBR	Unkn	Control	85.50
E09	SYBR	Unkn	Control	85.50
E10	SYBR	Unkn	CFG	85.50
E11	SYBR	Unkn	CFG	85.50
E12	SYBR	Unkn	CFG	86.00

Gene Expression

Analysis Mode: Normalized expression (ΔΔC(t))
Chart Data: Relative to zero
Scaling options:
Chart Error: ±1.0

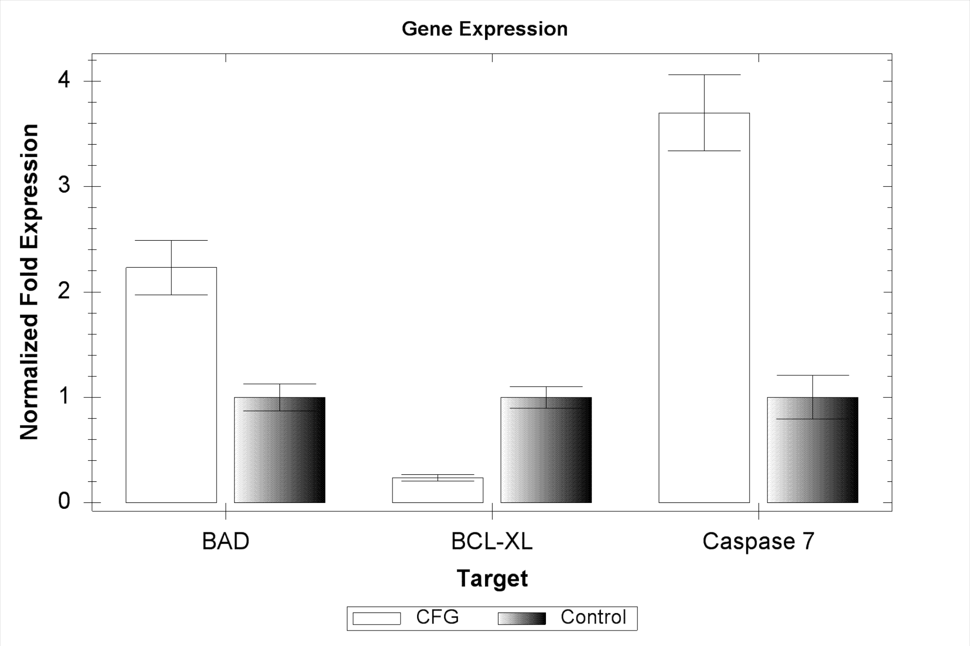

Target Names

Name	FullName	Reference	Auto Efficiency	Effiiciency
BAD	BAD	No	Yes	100.0%
BCL-XL	BCL-XL	No	Yes	100.0%
Caspase 7	Caspase 7	No	Yes	100.0%
GAPDH	GAPDH	Yes	Yes	100.0%

Sample Names

Name	FullName	Control
CFG	CFG	No
Control	Control	Yes

Gene Expression Data

Target	Sample	Ctrl	Expression	Expression SEM	Corrected Expression SEM	Mean C(t)	C(t) SEM
BAD	CFG		2.23198	0.25948	0.25948	23.78	0.14391
BAD	Control	*	1.00000	0.12791	0.12791	24.75	0.17757
BCL-XL	CFG		0.23598	0.03101	0.03101	23.80	0.16886
BCL-XL	Control	*	1.00000	0.10273	0.10273	21.53	0.13943
Caspase 7	CFG		3.69980	0.35967	0.35967	22.90	0.11068
Caspase 7	Control	*	1.00000	0.20703	0.20703	24.59	0.29442
GAPDH	CFG		N/A	N/A	N/A	19.31	0.08614
GAPDH	Control	*	N/A	N/A	N/A	19.12	0.05024

End Point

Fluorophore: SYBR
End Cycles to Average: 5
Mode: Percentage of Range - 10
Lowest RFU Value: 142
Highest RFU Value: 249
Cut Off Value:

End Point Data

Well	Fluor	Content	Sample	End RFU	Call
B07	SYBR	Unkn	Control	163	
B08	SYBR	Unkn	Control	142	
B09	SYBR	Unkn	Control	164	
B10	SYBR	Unkn	CFG	182	
B11	SYBR	Unkn	CFG	181	
B12	SYBR	Unkn	CFG	175	
C07	SYBR	Unkn	Control	232	
C08	SYBR	Unkn	Control	221	
C09	SYBR	Unkn	Control	234	
C10	SYBR	Unkn	CFG	179	
C11	SYBR	Unkn	CFG	162	
C12	SYBR	Unkn	CFG	167	
D07	SYBR	Unkn	Control	193	
D08	SYBR	Unkn	Control	196	
D09	SYBR	Unkn	Control	178	
D10	SYBR	Unkn	CFG	241	
D11	SYBR	Unkn	CFG	249	
D12	SYBR	Unkn	CFG	217	
E07	SYBR	Unkn	Control	189	
E08	SYBR	Unkn	Control	191	
E09	SYBR	Unkn	Control	208	
E10	SYBR	Unkn	CFG	182	
E11	SYBR	Unkn	CFG	185	
E12	SYBR	Unkn	CFG	176	
