# Supplementary material for: Fuling Granule, a Traditional Chinese Medicine Compound, Suppresses Cell Proliferation and TGFβ-Induced EMT in Ovarian Cancer
Source: PLoS One. 2016 Dec 30;11(12):e0168892. doi: 10.1371/journal.pone.0168892 (PMC5201296; doi:10.1371/journal.pone.0168892)
Supplement: S1 File — 01 GSE79454 and Gene Ontology Analysis 02 in vitro cell data:SRB, MTT, cell clony, cell distribution, wound healing, invasion and migration 03 qPCR data 04 in vivo data (ZIP) [file pone.0168892.s001.zip › supporting information/03QPCR data/SKOV3 Cell cycle QPCR/admin_2016-05-09 14-01-39_CC005309-2.pdf]

admin_2016-05-09 14-01-39_CC005309.pcrd

5/14/16 04:35 PM

Report Information

Experiment Date: 5/9/16 03:43 PM
User: BioRad\admin
Data File Name: admin_2016-05-09 14-01-39_CC005309.pcrd
Data File Path: G:\20160509
Selected Well Group: All Wells

Experiment Setup

Run Information

Run User: BioRad\admin
ID:
Notes:
Sample Volume: 25
Temperature Control Mode: Calculated
Lid Temperature: 105
Lid Force: AUTO

Protocol

1: 95.0°C for 1:00
2: 95.0°C for 0:20
3: 58.0°C for 0:20
4: 72.0°C for 0:20
Plate Read
5: GOTO 2, 39 more times
6: Melt Curve 65°C to 95°C : Increment 0.5°C for 0:05
Plate Read

Plate Display

	1	2	3	4	5	6	7	8	9	10	11	12
A	Unk<br>p27<br>Control	Unk<br>p27<br>Control	Unk<br>p27<br>Control	Unk<br>p27<br>CFG	Unk<br>p27<br>CFG	Unk<br>p27<br>CFG						
B	Unk<br>Cyclin E1<br>Control	Unk<br>Cyclin E1<br>Control	Unk<br>Cyclin E1<br>Control	Unk<br>Cyclin E1<br>CFG	Unk<br>Cyclin E1<br>CFG	Unk<br>Cyclin E1<br>CFG						
C	Unk<br>Cyclin D1<br>Control	Unk<br>Cyclin D1<br>Control	Unk<br>Cyclin D1<br>Control	Unk<br>Cyclin D1<br>CFG	Unk<br>Cyclin D1<br>CFG	Unk<br>Cyclin D1<br>CFG						
D	Unk<br>Cyclin B1<br>Control	Unk<br>Cyclin B1<br>Control	Unk<br>Cyclin B1<br>Control	Unk<br>Cyclin B1<br>CFG	Unk<br>Cyclin B1<br>CFG	Unk<br>Cyclin B1<br>CFG						
E	Unk<br>Cyclin A<br>Control	Unk<br>Cyclin A<br>Control	Unk<br>Cyclin A<br>Control	Unk<br>Cyclin A<br>CFG	Unk<br>Cyclin A<br>CFG	Unk<br>Cyclin A<br>CFG	Unk<br>GAPDH<br>Control	Unk<br>GAPDH<br>Control	Unk<br>GAPDH<br>Control	Unk<br>GAPDH<br>CFG	Unk<br>GAPDH<br>CFG	
F	Unk<br>CDK2<br>Control	Unk<br>CDK2<br>Control	Unk<br>CDK2<br>Control	Unk<br>CDK2<br>CFG	Unk<br>CDK2<br>CFG	Unk<br>CDK2<br>CFG						
G	Unk<br>CDK6<br>Control	Unk<br>CDK6<br>Control	Unk<br>CDK6<br>Control	Unk<br>CDK6<br>CFG	Unk<br>CDK6<br>CFG	Unk<br>CDK6<br>CFG						
H	Unk<br>E2F1<br>Control	Unk<br>E2F1<br>Control	Unk<br>E2F1<br>Control	Unk<br>E2F1<br>CFG	Unk<br>E2F1<br>CFG	Unk<br>E2F1<br>CFG						

Quantitation

Step #: 4
Analysis Mode: Baseline Subtracted Curve Fit
Baseline Method per Fluorophore:
SYBR: Auto Calculated
Threshold Setting per Fluorophore:
SYBR: 10.30, Auto Calculated

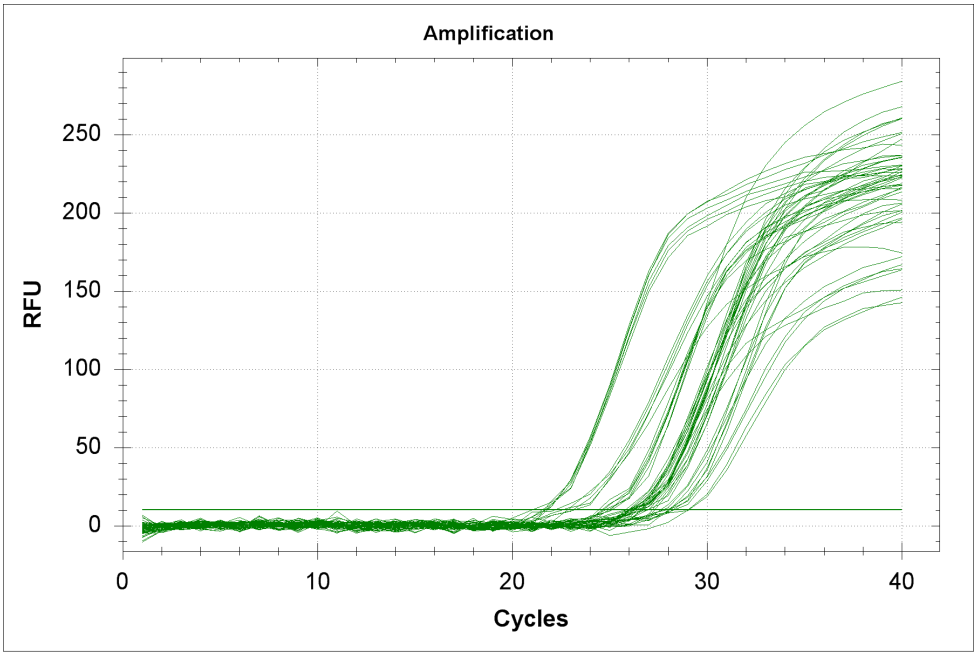

Quantitation Data

Well	Fluor	Content	Target	Sample	Threshold Cycle (C(t))	C(t) Mean	C(t) Std. Dev
A01	SYBR	Unkn	p27	Control	29.03	29.03	0.000
A02	SYBR	Unkn	p27	Control	29.07	29.07	0.000
A03	SYBR	Unkn	p27	Control	27.74	27.74	0.000
A04	SYBR	Unkn	p27	CFG	28.33	28.33	0.000
A05	SYBR	Unkn	p27	CFG	27.95	27.95	0.000
A06	SYBR	Unkn	p27	CFG	26.15	26.15	0.000
B01	SYBR	Unkn	Cyclin E1	Control	27.62	27.62	0.000
B02	SYBR	Unkn	Cyclin E1	Control	28.08	28.08	0.000
B03	SYBR	Unkn	Cyclin E1	Control	27.19	27.19	0.000
B04	SYBR	Unkn	Cyclin E1	CFG	28.08	28.08	0.000
B05	SYBR	Unkn	Cyclin E1	CFG	28.25	28.25	0.000
B06	SYBR	Unkn	Cyclin E1	CFG	28.04	28.04	0.000
C01	SYBR	Unkn	Cyclin D1	Control	25.96	25.96	0.000
C02	SYBR	Unkn	Cyclin D1	Control	26.30	26.30	0.000
C03	SYBR	Unkn	Cyclin D1	Control	26.04	26.04	0.000
C04	SYBR	Unkn	Cyclin D1	CFG	25.89	25.89	0.000
C05	SYBR	Unkn	Cyclin D1	CFG	26.24	26.24	0.000
C06	SYBR	Unkn	Cyclin D1	CFG	25.83	25.83	0.000
D01	SYBR	Unkn	Cyclin B1	Control	21.05	21.05	0.000
D02	SYBR	Unkn	Cyclin B1	Control	21.56	21.56	0.000
D03	SYBR	Unkn	Cyclin B1	Control	21.70	21.70	0.000
D04	SYBR	Unkn	Cyclin B1	CFG	21.81	21.81	0.000
D05	SYBR	Unkn	Cyclin B1	CFG	21.72	21.72	0.000
D06	SYBR	Unkn	Cyclin B1	CFG	21.65	21.65	0.000
E01	SYBR	Unkn	Cyclin A	Control	26.72	26.72	0.000
E02	SYBR	Unkn	Cyclin A	Control	25.11	25.11	0.000
E03	SYBR	Unkn	Cyclin A	Control	26.64	26.64	0.000
E04	SYBR	Unkn	Cyclin A	CFG	27.31	27.31	0.000
E05	SYBR	Unkn	Cyclin A	CFG	27.01	27.01	0.000
E06	SYBR	Unkn	Cyclin A	CFG	26.64	26.64	0.000
E07	SYBR	Unkn	GAPDH	Control	23.21	23.21	0.000
E08	SYBR	Unkn	GAPDH	Control	22.21	22.21	0.000
E09	SYBR	Unkn	GAPDH	Control	22.86	22.86	0.000
E10	SYBR	Unkn	GAPDH	CFG	23.12	23.12	0.000
E11	SYBR	Unkn	GAPDH	CFG	22.61	22.61	0.000
F01	SYBR	Unkn	CDK2	Control	26.26	26.26	0.000
F02	SYBR	Unkn	CDK2	Control	26.02	26.02	0.000
F03	SYBR	Unkn	CDK2	Control	26.57	26.57	0.000
F04	SYBR	Unkn	CDK2	CFG	26.11	26.11	0.000
F05	SYBR	Unkn	CDK2	CFG	26.95	26.95	0.000
F06	SYBR	Unkn	CDK2	CFG	26.47	26.47	0.000

G01	SYBR	Unkn	CDK6	Control	25.06	25.06	0.000
G02	SYBR	Unkn	CDK6	Control	24.78	24.78	0.000
G03	SYBR	Unkn	CDK6	Control	24.44	24.44	0.000
G04	SYBR	Unkn	CDK6	CFG	24.95	24.95	0.000
G05	SYBR	Unkn	CDK6	CFG	24.68	24.68	0.000
G06	SYBR	Unkn	CDK6	CFG	24.61	24.61	0.000
H01	SYBR	Unkn	E2F1	Control	25.98	25.98	0.000
H02	SYBR	Unkn	E2F1	Control	26.05	26.05	0.000
H03	SYBR	Unkn	E2F1	Control	26.11	26.11	0.000
H04	SYBR	Unkn	E2F1	CFG	26.25	26.25	0.000
H05	SYBR	Unkn	E2F1	CFG	26.41	26.41	0.000
H06	SYBR	Unkn	E2F1	CFG	26.24	26.24	0.000

Melt Curve

Step #: 6
Threshold bar settings:

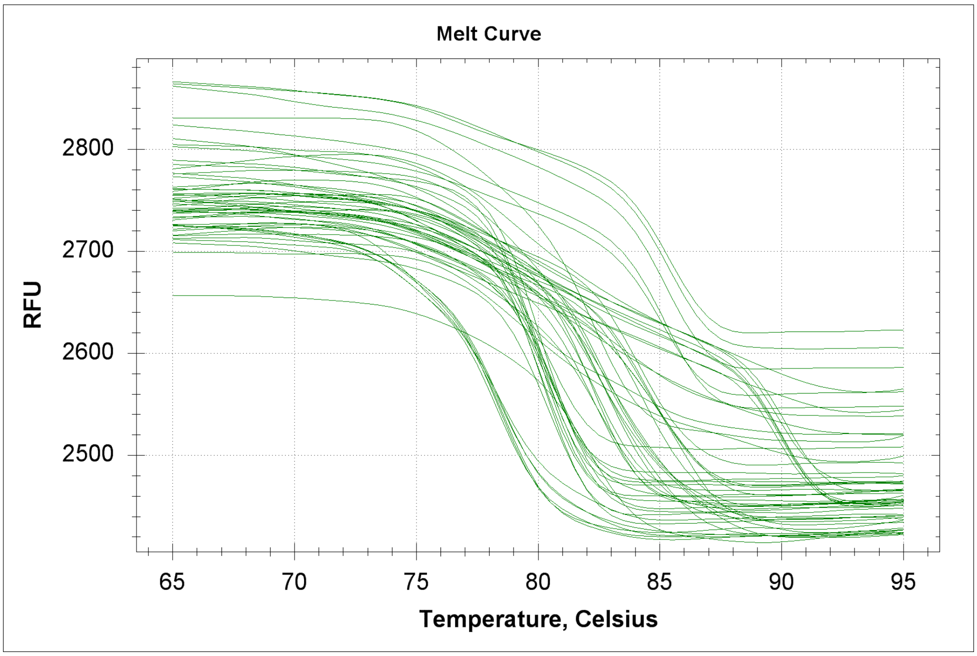

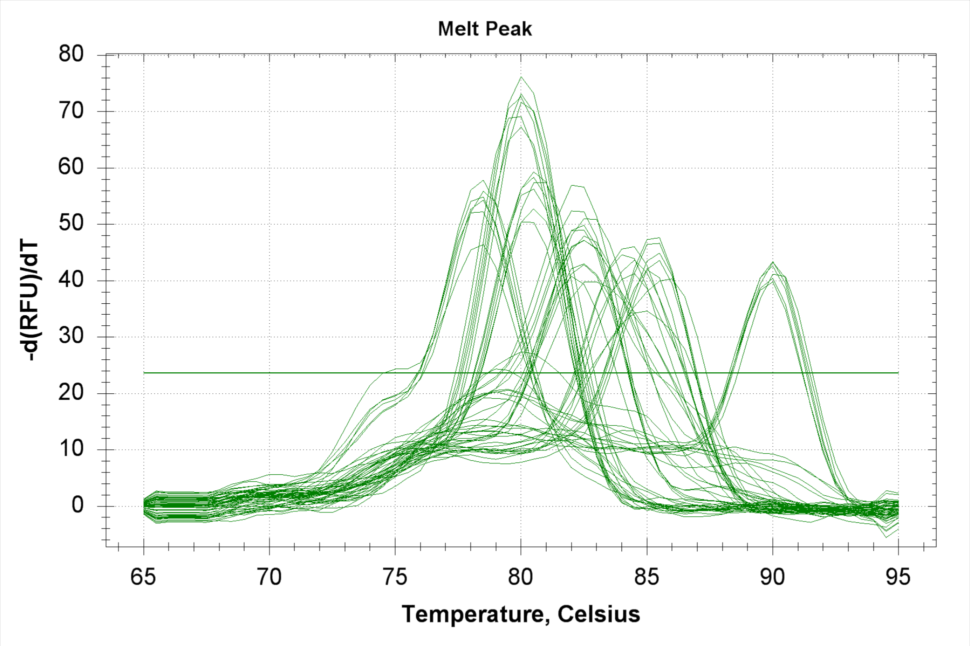

Melt Curve Data

Well	Fluor	Content	Sample	Melt Temp
A02	SYBR	Unkn	Control	79.00

A03	SYBR	Unkn	Control	80.00
B01	SYBR	Unkn	Control	85.00
B02	SYBR	Unkn	Control	84.50
B03	SYBR	Unkn	Control	86.00
B04	SYBR	Unkn	CFG	84.50
B05	SYBR	Unkn	CFG	84.50
B06	SYBR	Unkn	CFG	84.00
C01	SYBR	Unkn	Control	81.00
C02	SYBR	Unkn	Control	80.50
C03	SYBR	Unkn	Control	80.50
C04	SYBR	Unkn	CFG	80.50
C05	SYBR	Unkn	CFG	80.00
C06	SYBR	Unkn	CFG	80.50
D01	SYBR	Unkn	Control	80.00
D02	SYBR	Unkn	Control	80.00
D03	SYBR	Unkn	Control	80.00
D04	SYBR	Unkn	CFG	80.00
D05	SYBR	Unkn	CFG	80.00
D06	SYBR	Unkn	CFG	80.00
E01	SYBR	Unkn	Control	82.50
E02	SYBR	Unkn	Control	82.00
E03	SYBR	Unkn	Control	82.50
E04	SYBR	Unkn	CFG	82.50
E05	SYBR	Unkn	CFG	82.00
E06	SYBR	Unkn	CFG	82.00
E07	SYBR	Unkn	Control	85.00
E08	SYBR	Unkn	Control	85.50
E09	SYBR	Unkn	Control	85.50
E10	SYBR	Unkn	CFG	85.50
E11	SYBR	Unkn	CFG	85.50
F01	SYBR	Unkn	Control	82.50
F02	SYBR	Unkn	Control	82.50
F03	SYBR	Unkn	Control	82.50
F04	SYBR	Unkn	CFG	82.50
F05	SYBR	Unkn	CFG	82.50
F06	SYBR	Unkn	CFG	82.50
G01	SYBR	Unkn	Control	78.50
G02	SYBR	Unkn	Control	78.50
G03	SYBR	Unkn	Control	78.50
G04	SYBR	Unkn	CFG	78.50
G05	SYBR	Unkn	CFG	78.50
G06	SYBR	Unkn	CFG	78.50
H01	SYBR	Unkn	Control	90.00
H02	SYBR	Unkn	Control	90.00
H03	SYBR	Unkn	Control	90.00
H04	SYBR	Unkn	CFG	90.00
H05	SYBR	Unkn	CFG	90.00
H06	SYBR	Unkn	CFG	90.00

Gene Expression

Analysis Mode: Normalized expression (ΔΔC(t))
Chart Data: Relative to zero
Scaling options:
Chart Error: ±1.0

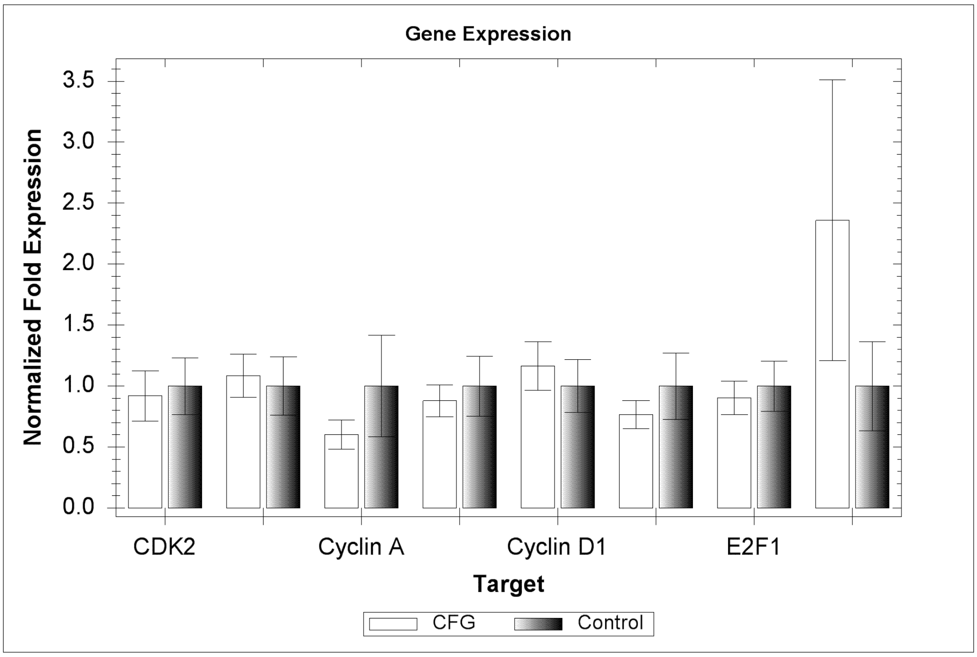

Target Names

Name	FullName	Reference	Auto Efficiency	Effiiciency
CDK2	CDK2	No	Yes	100.0%
CDK6	CDK6	No	Yes	100.0%
Cyclin A	Cyclin A	No	Yes	100.0%
Cyclin B1	Cyclin B1	No	Yes	100.0%
Cyclin D1	Cyclin D1	No	Yes	100.0%
Cyclin E1	Cyclin E1	No	Yes	100.0%
E2F1	E2F1	No	Yes	100.0%
GAPDH	GAPDH	Yes	Yes	100.0%
p27	p27	No	Yes	100.0%

Sample Names

Name	FullName	Control
CFG	CFG	No
Control	Control	Yes

Gene Expression Data

Target	Sample	Ctrl	Expression	Expression SEM	Corrected Expression SEM	Mean C(t)	C(t) SEM
CDK2	CFG		0.92011	0.20544	0.20544	26.51	0.24430
CDK2	Control	*	1.00000	0.23209	0.23209	26.29	0.15844
CDK6	CFG		1.08497	0.17574	0.17574	24.75	0.10260
CDK6	Control	*	1.00000	0.23952	0.23952	24.76	0.17998
Cyclin A	CFG		0.60367	0.11996	0.11996	26.99	0.19523
Cyclin A	Control	*	1.00000	0.41655	0.41655	26.16	0.52358
Cyclin B1	CFG		0.87974	0.13122	0.13122	21.73	0.04719
Cyclin B1	Control	*	1.00000	0.24620	0.24620	21.44	0.19786
Cyclin D1	CFG		1.16508	0.19834	0.19834	25.98	0.12743
Cyclin D1	Control	*	1.00000	0.21658	0.21658	26.10	0.10306
Cyclin E1	CFG		0.76499	0.11627	0.11627	28.12	0.06326
Cyclin E1	Control	*	1.00000	0.27205	0.27205	27.63	0.25891
E2F1	CFG		0.90288	0.13621	0.13621	26.30	0.05736
E2F1	Control	*	1.00000	0.20620	0.20620	26.05	0.03853
GAPDH	CFG		N/A	N/A	N/A	22.86	0.25713
GAPDH	Control	*	N/A	N/A	N/A	22.76	0.29497
p27	CFG		2.35876	1.15067	1.15067	27.48	0.67174
p27	Control	*	1.00000	0.36585	0.36585	28.61	0.43769

End Point

Fluorophore: SYBR
End Cycles to Average: 5
Mode: Percentage of Range - 10

Lowest RFU Value: 136
Highest RFU Value: 275
Cut Off Value:

End Point Data

Well	Fluor	Content	Sample	End RFU	Call
A01	SYBR	Unkn	Control	136	
A02	SYBR	Unkn	Control	136	
A03	SYBR	Unkn	Control	163	
A04	SYBR	Unkn	CFG	156	
A05	SYBR	Unkn	CFG	157	
A06	SYBR	Unkn	CFG	146	
B01	SYBR	Unkn	Control	187	
B02	SYBR	Unkn	Control	206	
B03	SYBR	Unkn	Control	212	
B04	SYBR	Unkn	CFG	214	
B05	SYBR	Unkn	CFG	191	
B06	SYBR	Unkn	CFG	189	
C01	SYBR	Unkn	Control	203	
C02	SYBR	Unkn	Control	185	
C03	SYBR	Unkn	Control	214	
C04	SYBR	Unkn	CFG	197	
C05	SYBR	Unkn	CFG	155	
C06	SYBR	Unkn	CFG	193	
D01	SYBR	Unkn	Control	224	
D02	SYBR	Unkn	Control	245	
D03	SYBR	Unkn	Control	233	
D04	SYBR	Unkn	CFG	241	
D05	SYBR	Unkn	CFG	224	
D06	SYBR	Unkn	CFG	228	
E01	SYBR	Unkn	Control	217	
E02	SYBR	Unkn	Control	275	
E03	SYBR	Unkn	Control	228	
E04	SYBR	Unkn	CFG	227	
E05	SYBR	Unkn	CFG	189	
E06	SYBR	Unkn	CFG	251	
E07	SYBR	Unkn	Control	177	
E08	SYBR	Unkn	Control	224	
E09	SYBR	Unkn	Control	224	
E10	SYBR	Unkn	CFG	215	
E11	SYBR	Unkn	CFG	208	
F01	SYBR	Unkn	Control	240	
F02	SYBR	Unkn	Control	248	
F03	SYBR	Unkn	Control	249	
F04	SYBR	Unkn	CFG	257	
F05	SYBR	Unkn	CFG	231	
F06	SYBR	Unkn	CFG	230	
G01	SYBR	Unkn	Control	215	
G02	SYBR	Unkn	Control	216	
G03	SYBR	Unkn	Control	205	
G04	SYBR	Unkn	CFG	213	
G05	SYBR	Unkn	CFG	217	
G06	SYBR	Unkn	CFG	211	
H01	SYBR	Unkn	Control	223	
H02	SYBR	Unkn	Control	228	
H03	SYBR	Unkn	Control	225	
H04	SYBR	Unkn	CFG	218	
H05	SYBR	Unkn	CFG	213	
H06	SYBR	Unkn	CFG	198	
