# Supplementary figures and images for: Correction: Fuling Granule, a Traditional Chinese Medicine Compound, Suppresses Cell Proliferation and TGFβ-Induced EMT in Ovarian Cancer
Source: PLoS One. 2023 Dec 21;18(12):e0296410. doi: 10.1371/journal.pone.0296410 (PMC10734952; doi:10.1371/journal.pone.0296410)

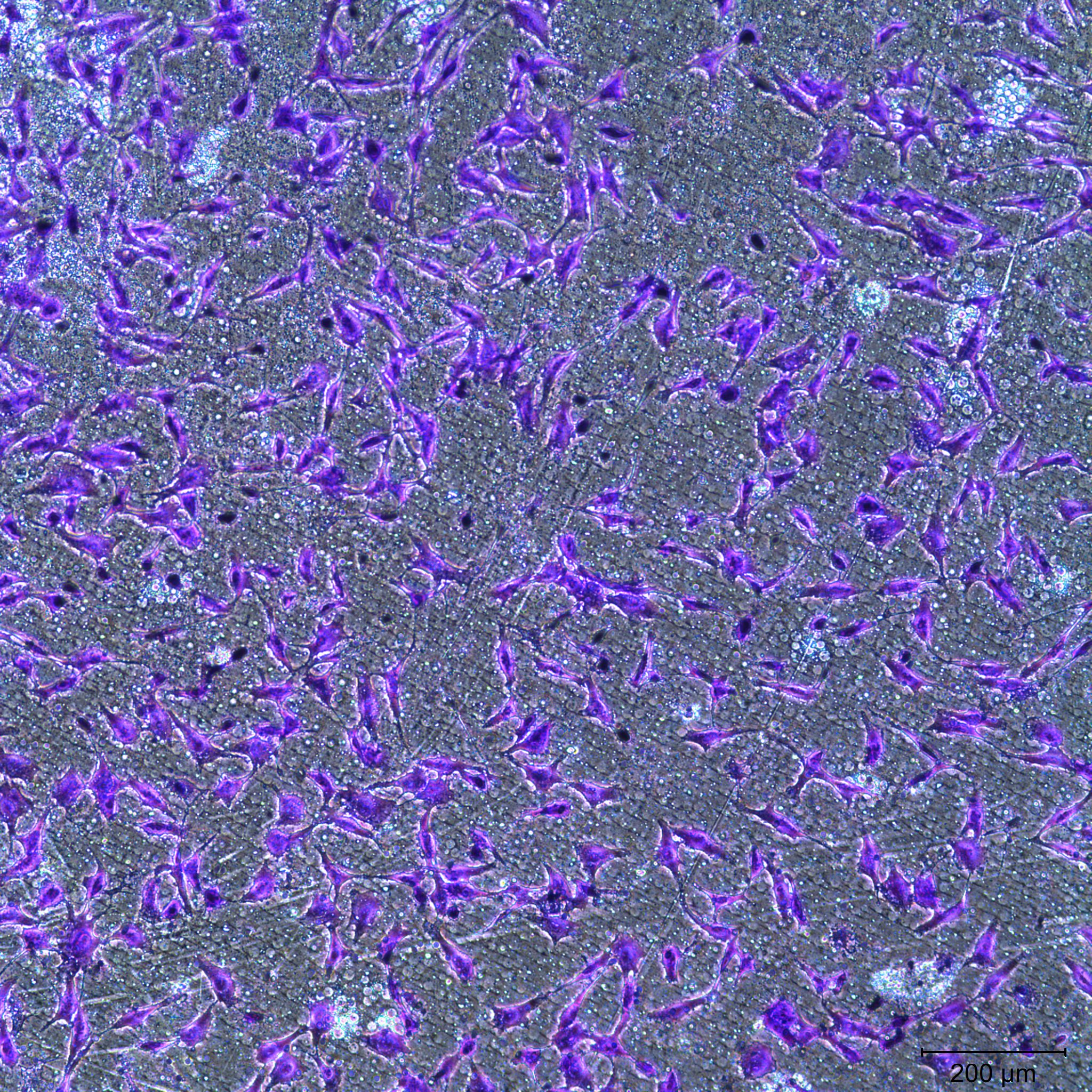

Supplement: S1 File — (ZIP) [file pone.0296410.s001.zip › HEY cells invasion images/Mock group/Image1.jpg]

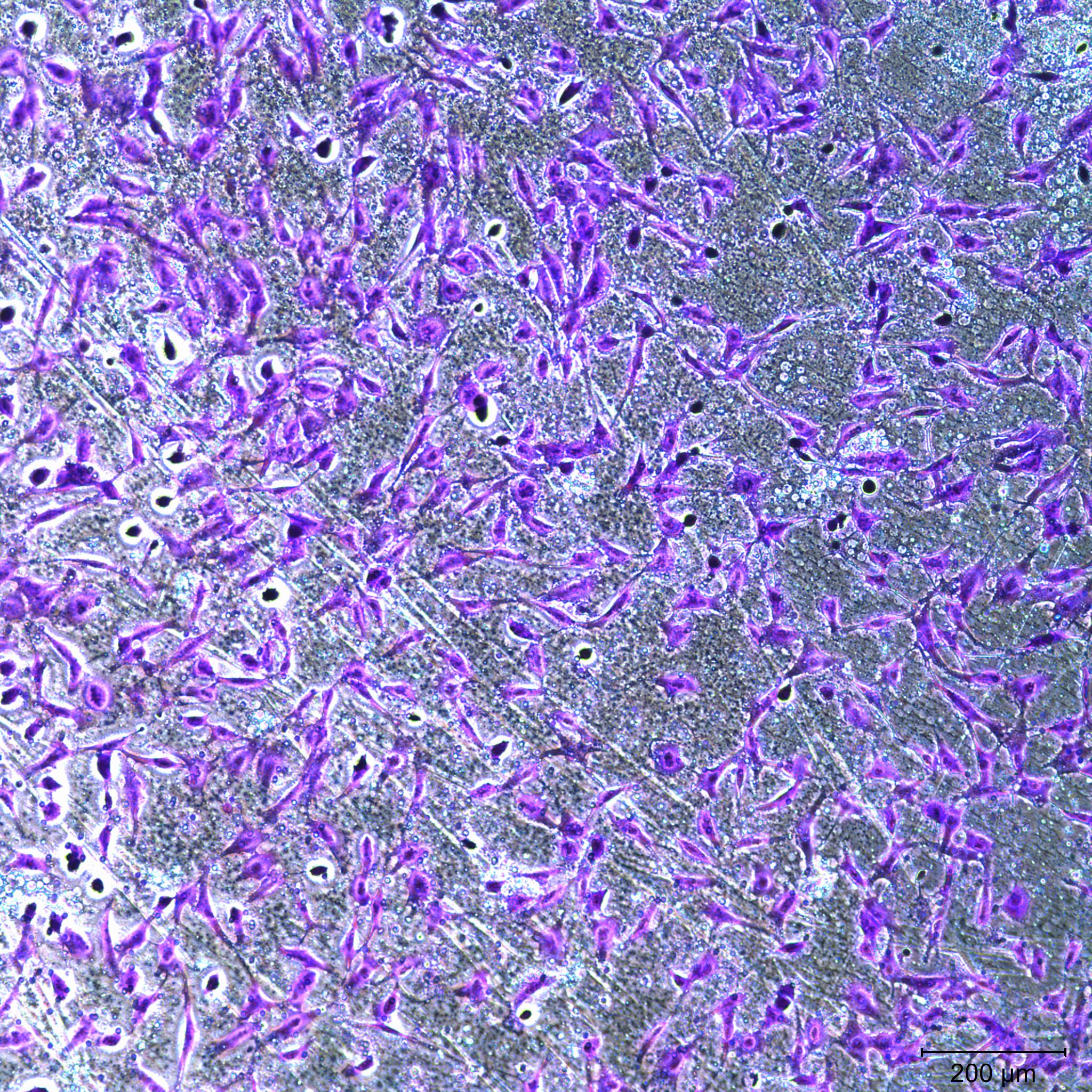

Supplement: S1 File — (ZIP) [file pone.0296410.s001.zip › HEY cells invasion images/Mock group/Image2.jpg]

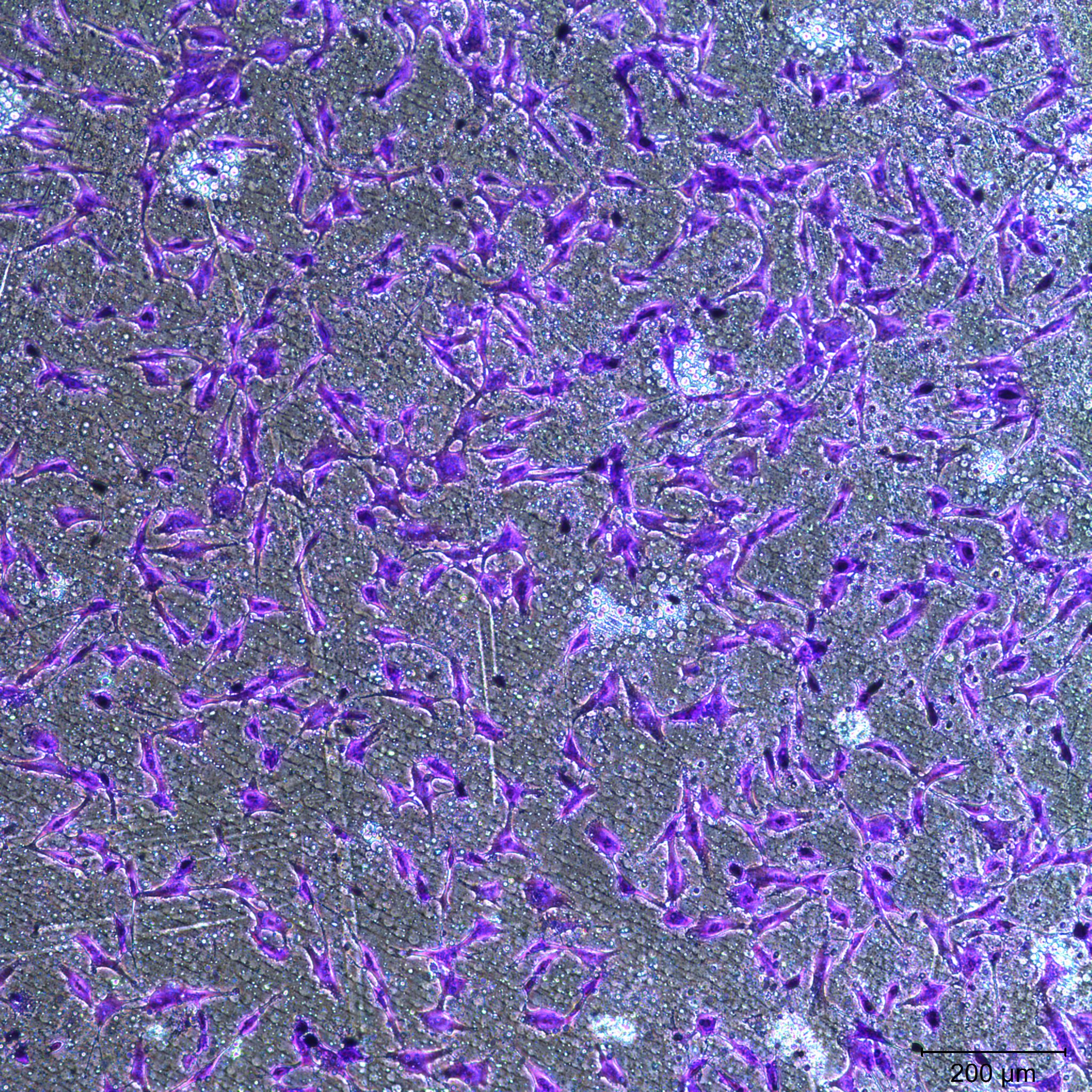

Supplement: S1 File — (ZIP) [file pone.0296410.s001.zip › HEY cells invasion images/Mock group/Image3.jpg]

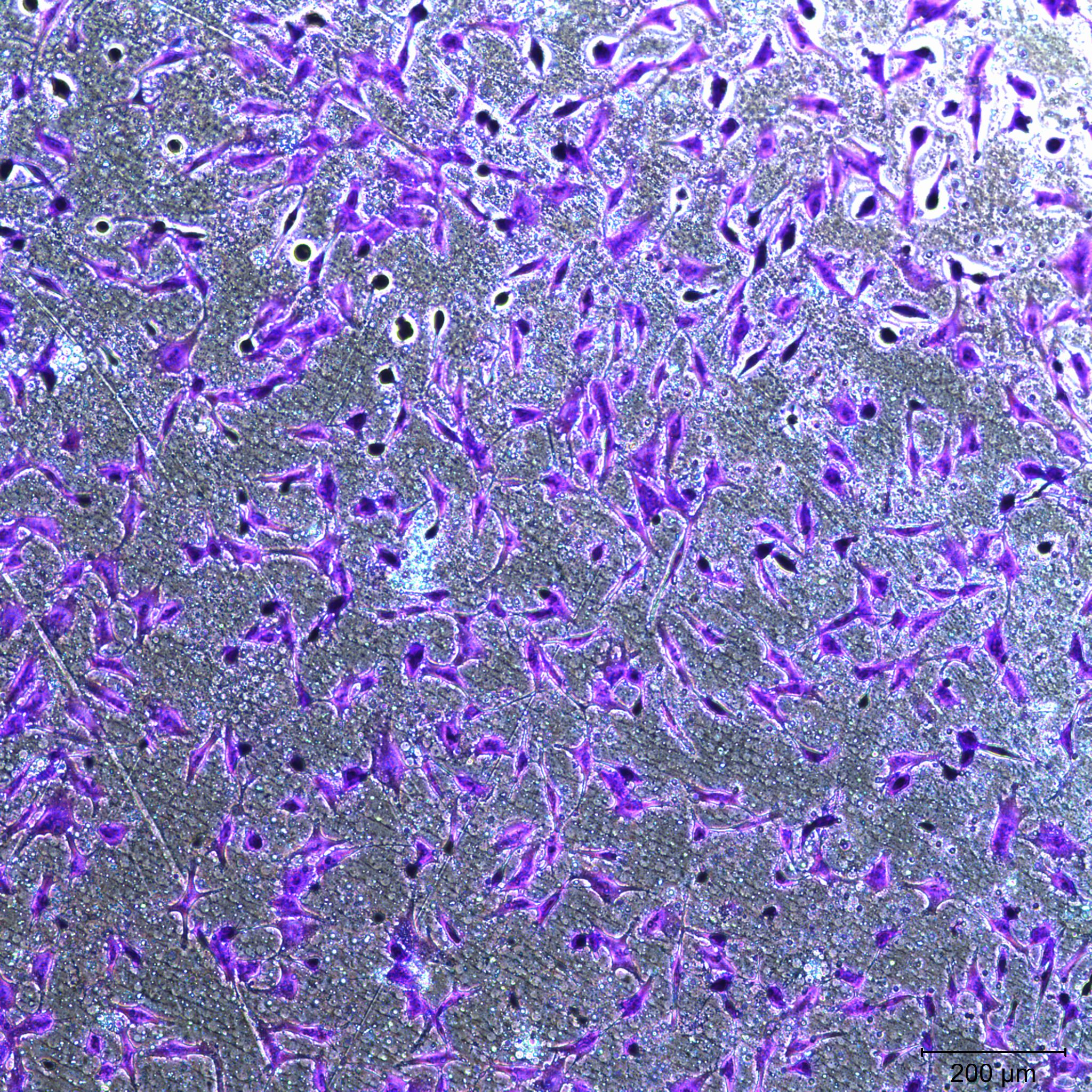

Supplement: S1 File — (ZIP) [file pone.0296410.s001.zip › HEY cells invasion images/Mock group/Image4.jpg]

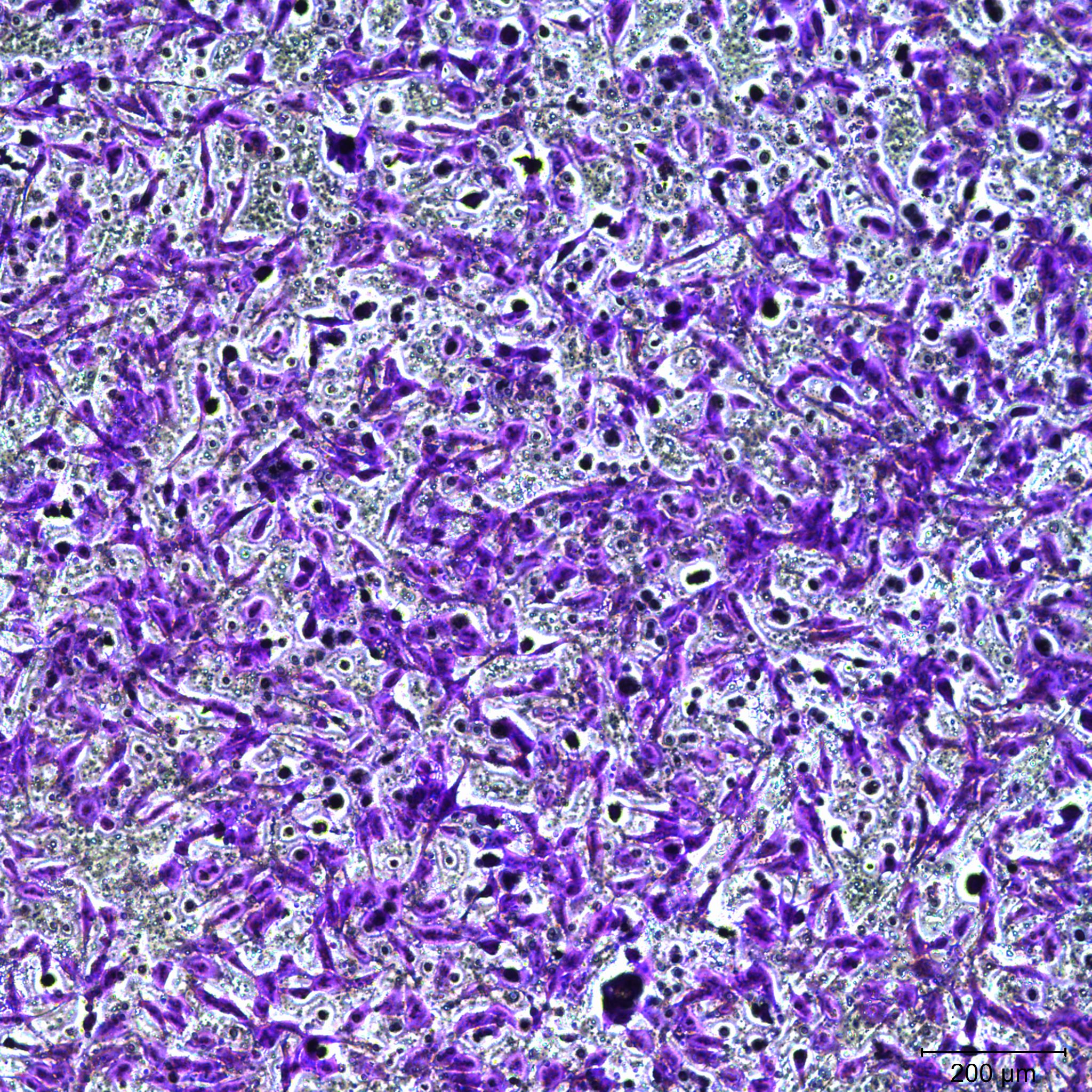

Supplement: S1 File — (ZIP) [file pone.0296410.s001.zip › HEY cells invasion images/TGFβ1 group/Image1.jpg]

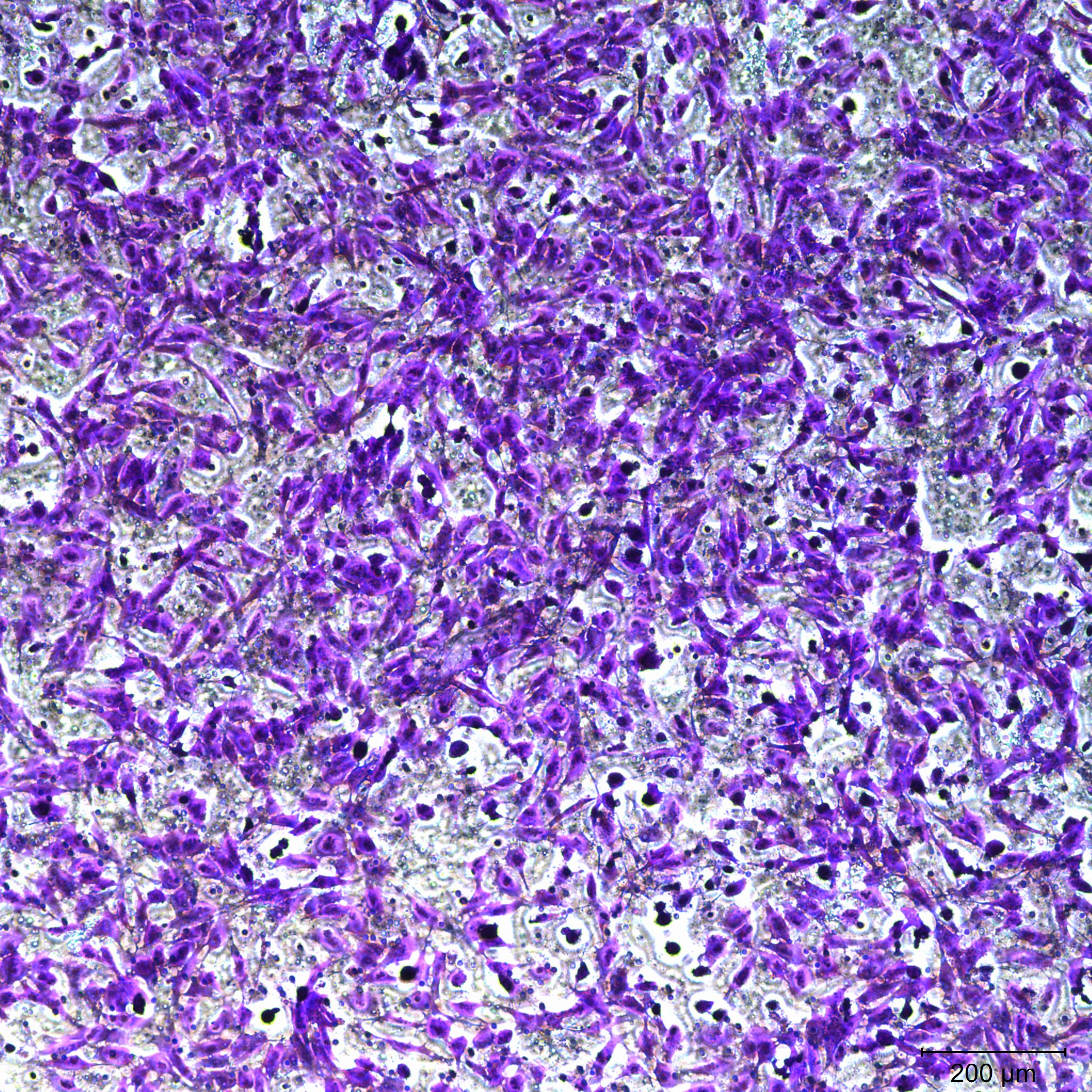

Supplement: S1 File — (ZIP) [file pone.0296410.s001.zip › HEY cells invasion images/TGFβ1 group/Image2.jpg]

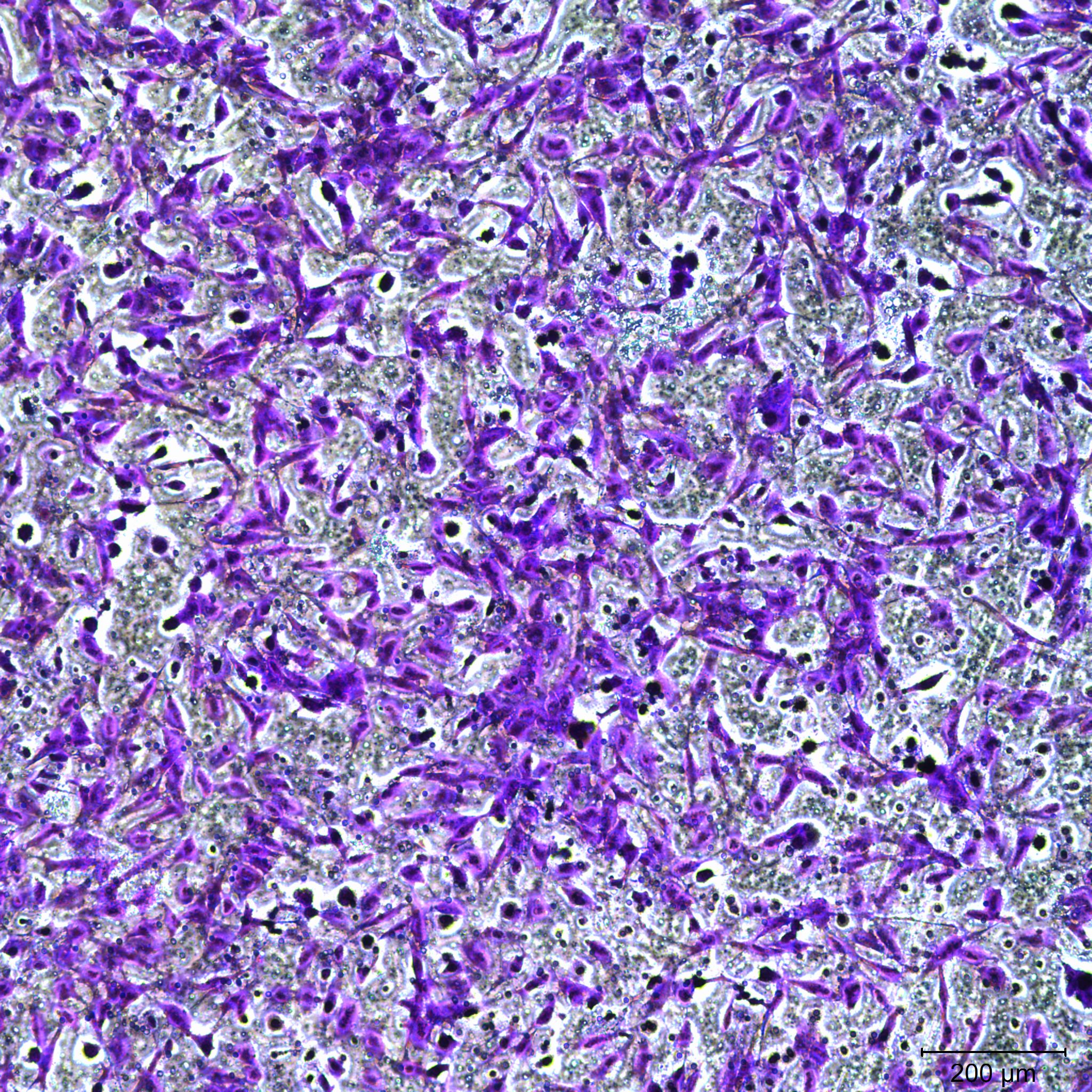

Supplement: S1 File — (ZIP) [file pone.0296410.s001.zip › HEY cells invasion images/TGFβ1 group/Image3.jpg]

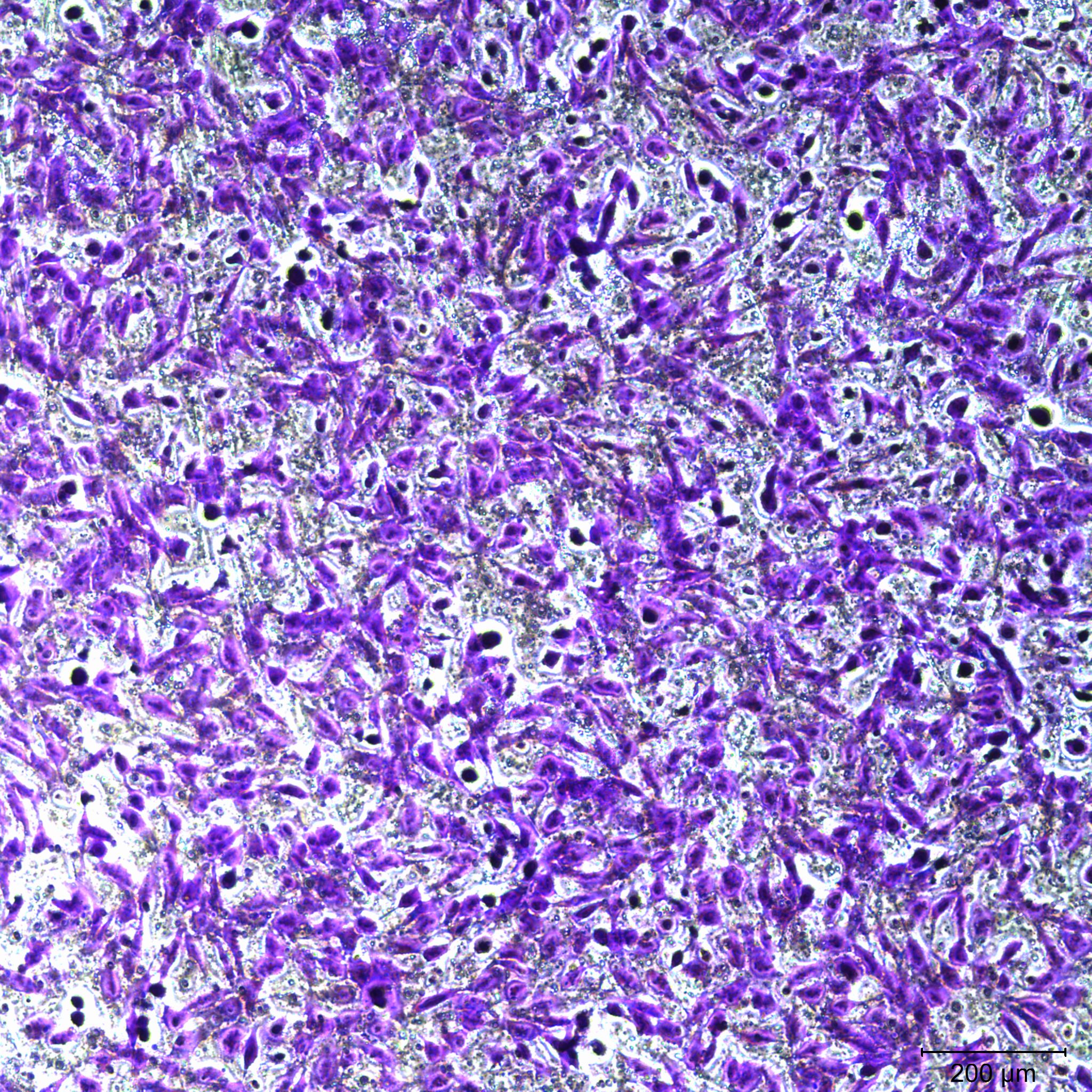

Supplement: S1 File — (ZIP) [file pone.0296410.s001.zip › HEY cells invasion images/TGFβ1 group/Image4.jpg]

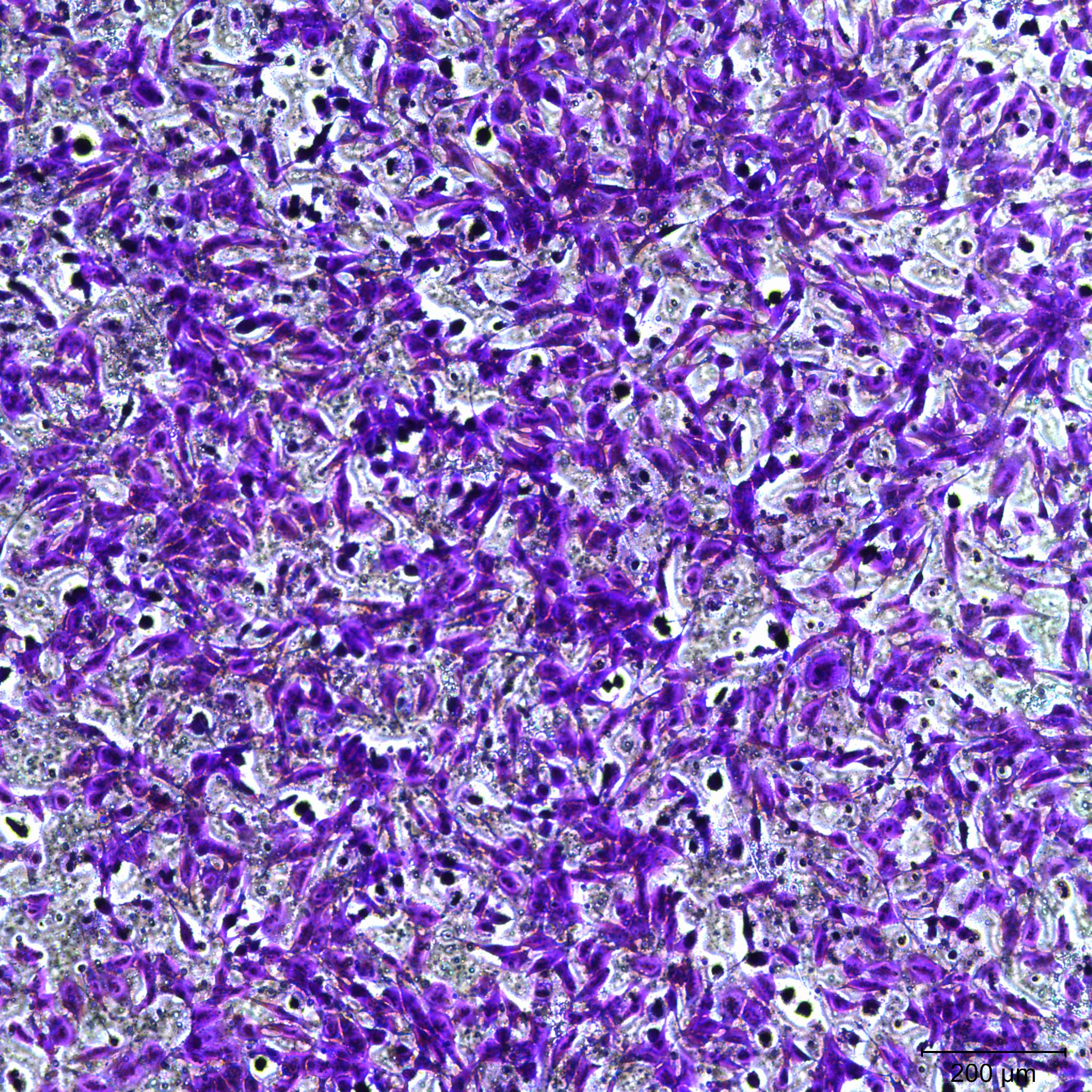

Supplement: S1 File — (ZIP) [file pone.0296410.s001.zip › HEY cells invasion images/TGFβ1 group/Image5.jpg]

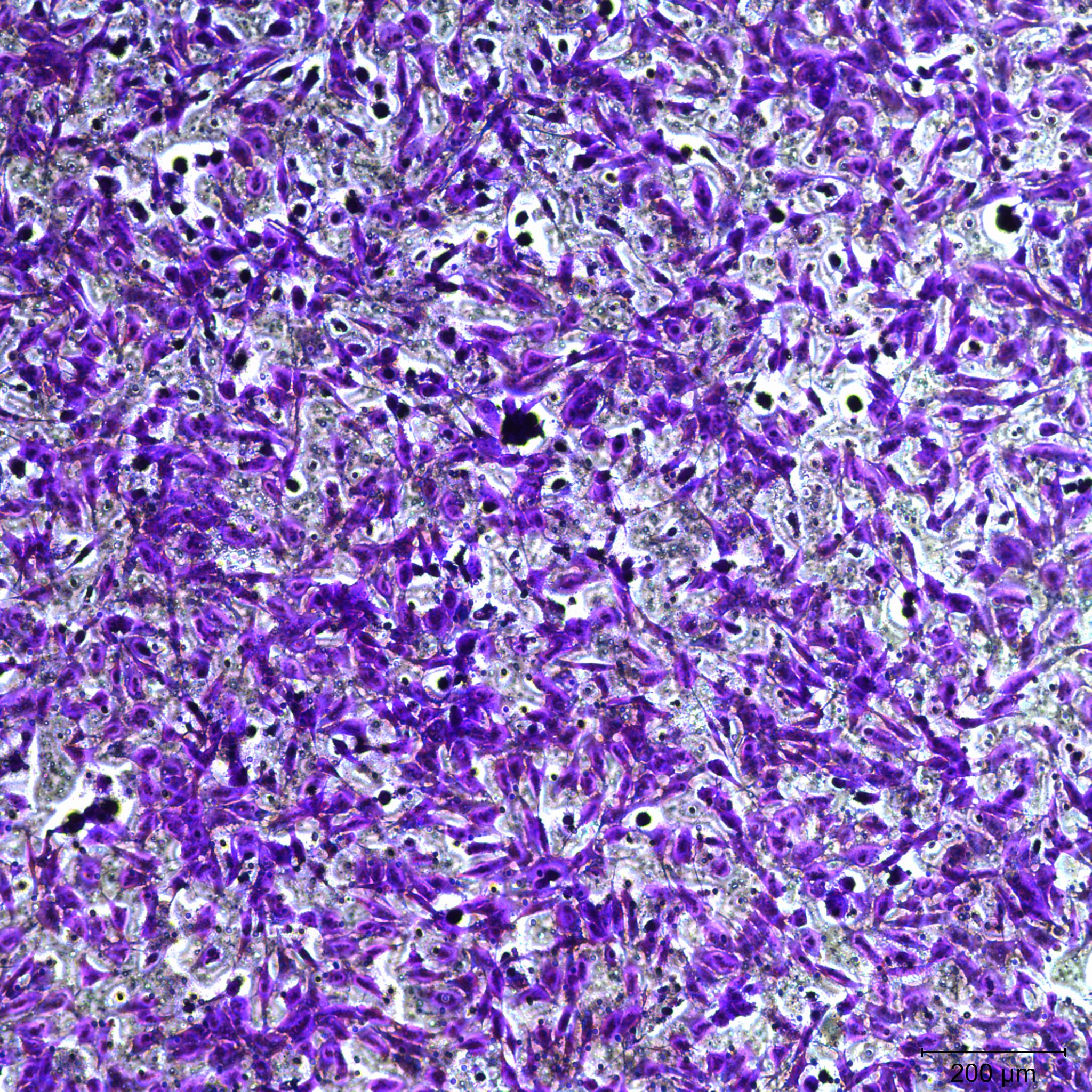

Supplement: S1 File — (ZIP) [file pone.0296410.s001.zip › HEY cells invasion images/TGFβ1 group/Image6.jpg]

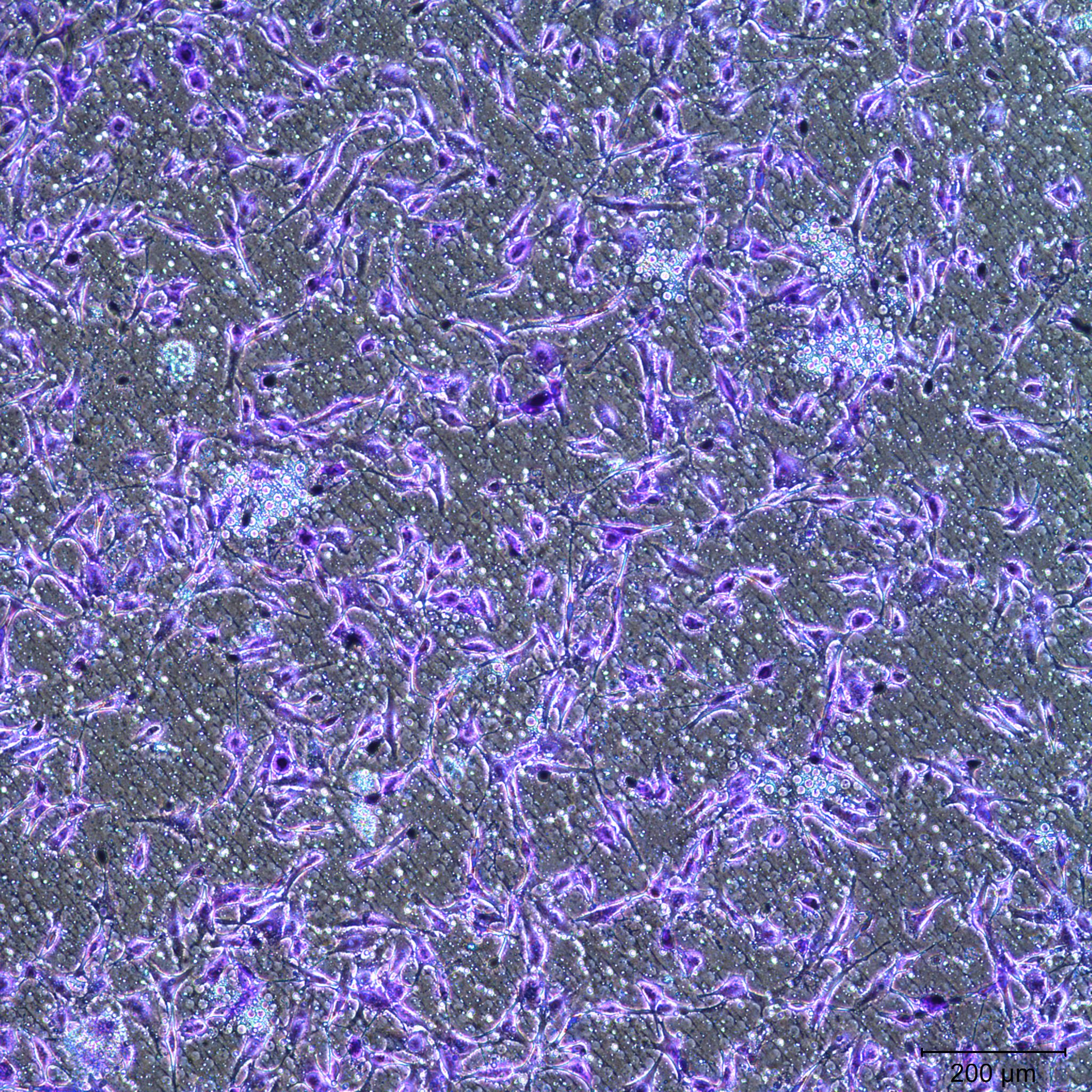

Supplement: S1 File — (ZIP) [file pone.0296410.s001.zip › HEY cells invasion images/TGFβ1+CFG group/Image1.jpg]

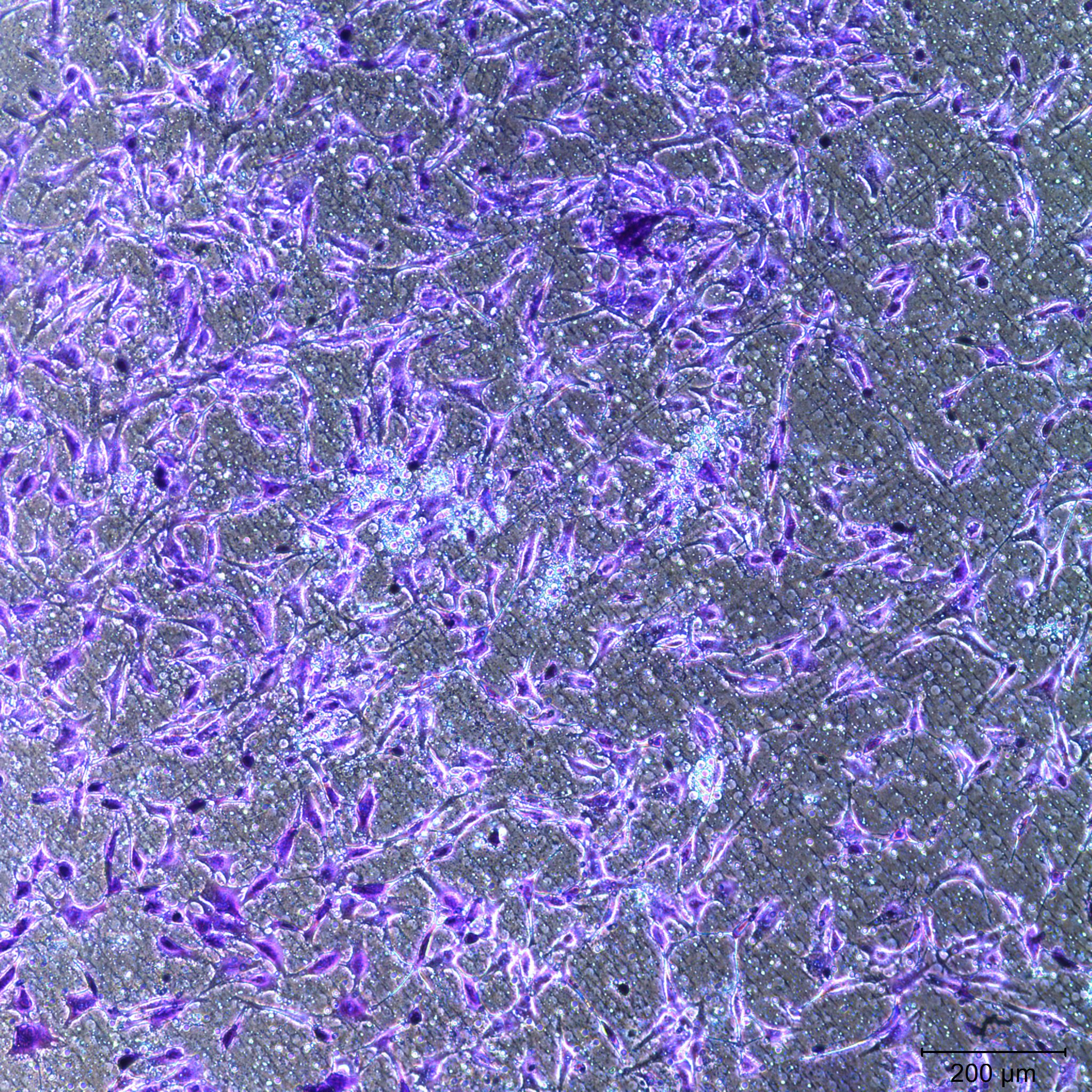

Supplement: S1 File — (ZIP) [file pone.0296410.s001.zip › HEY cells invasion images/TGFβ1+CFG group/Image2.jpg]

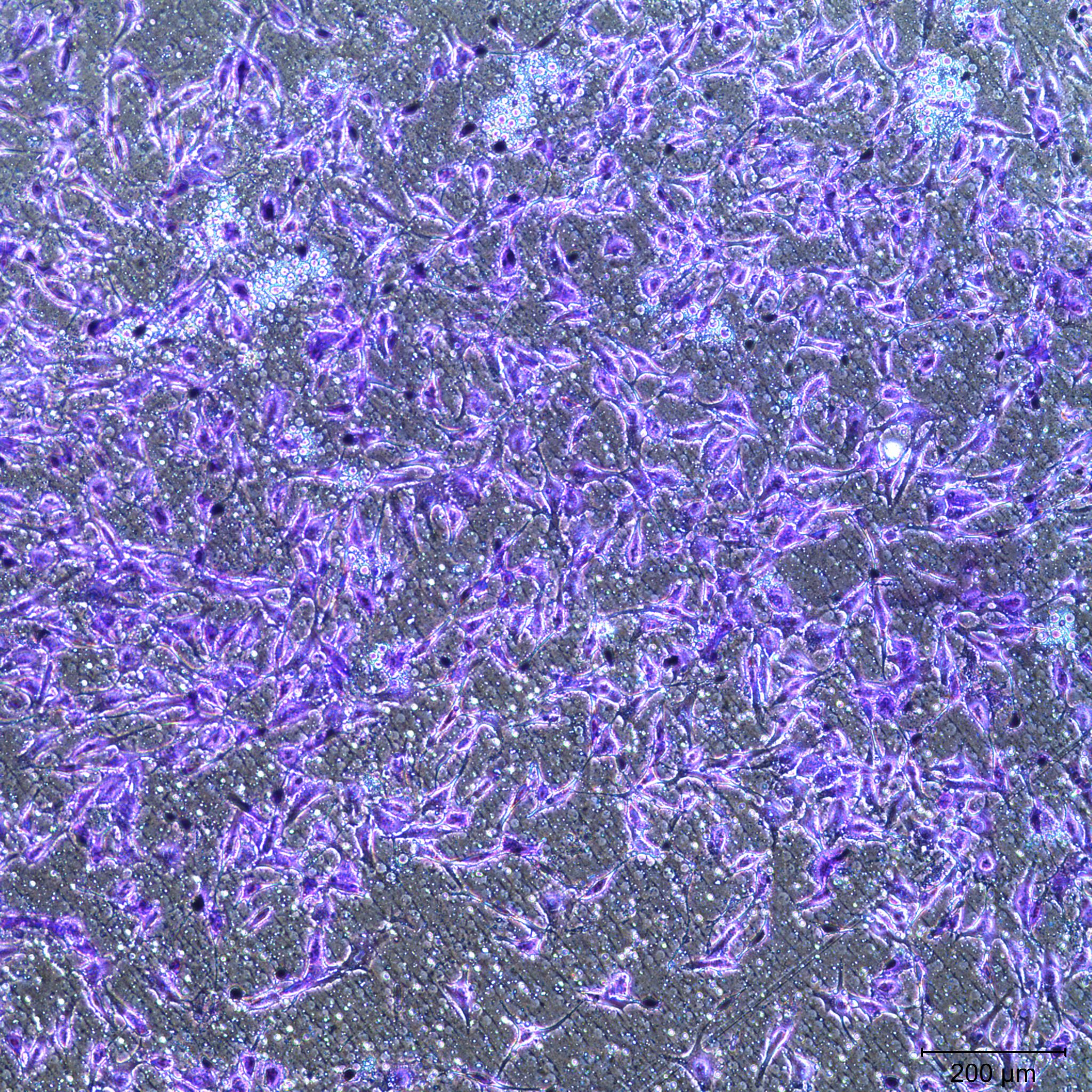

Supplement: S1 File — (ZIP) [file pone.0296410.s001.zip › HEY cells invasion images/TGFβ1+CFG group/Image3.jpg]

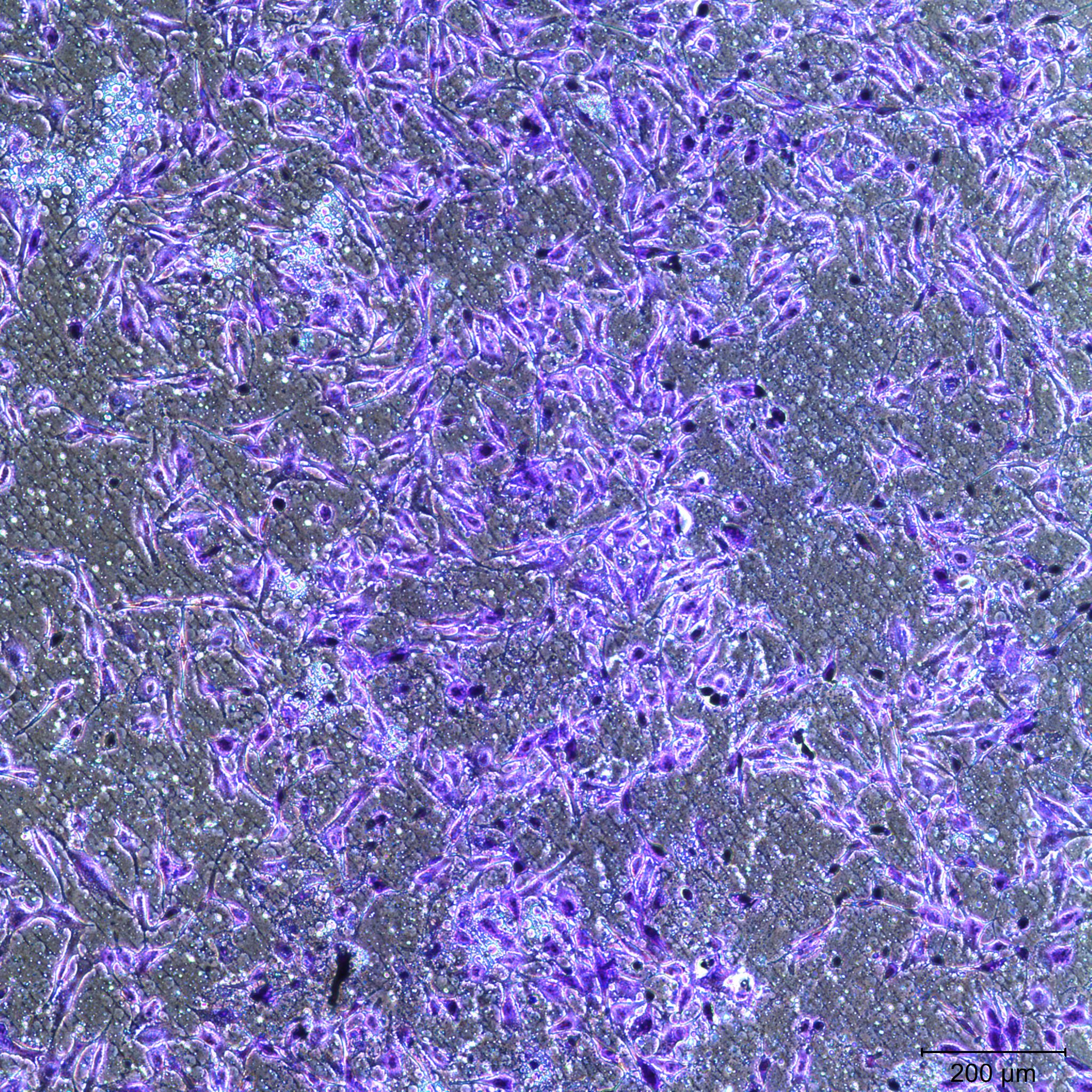

Supplement: S1 File — (ZIP) [file pone.0296410.s001.zip › HEY cells invasion images/TGFβ1+CFG group/Image4.jpg]
